# Interleukin-17A pathway target genes are upregulated in *Equus caballus* supporting limb laminitis

**DOI:** 10.1371/journal.pone.0232920

**Published:** 2020-12-10

**Authors:** Lynne Cassimeris, Julie B. Engiles, Hannah Galantino-Homer

**Affiliations:** 1 Department of Biological Sciences, Lehigh University, Bethlehem, Pennsylvania, United States of America; 2 Department of Clinical Studies/New Bolton Center, School of Veterinary Medicine, University of Pennsylvania, Kennett Square, Pennsylvania, United States of America; 3 Department of Pathobiology, School of Veterinary Medicine, University of Pennsylvania, Philadelphia, Pennsylvania, United States of America; University of Bern, SWITZERLAND

## Abstract

Supporting Limb Laminitis (SLL) is a painful and crippling secondary complication of orthopedic injuries and infections in horses, often resulting in euthanasia. SLL causes structural alterations and inflammation of the interdigitating layers of specialized epidermal and dermal tissues, the lamellae, which suspend the equine distal phalanx from the hoof capsule. Activation of the interleukin-17A (IL-17A)-dependent inflammatory pathway is an epidermal stress response that contributes to physiologic cutaneous wound healing as well as pathological skin conditions. As a first test of the hypothesis that hoof lamellae of horses diagnosed with SLL also respond to stress by activating the IL-17A pathway, the expression of IL-17A, IL-17 receptor subunit A and 11 IL-17A effector genes was measured by RT-PCR or qPCR. Lamellar tissue was isolated from Thoroughbreds euthanized due to naturally occurring SLL and in age and breed matched non-laminitic controls. By RT-PCR, the IL-17 Receptor A subunit was expressed in both non-laminitic and laminitic tissues, while IL-17A was primarily detectable in laminitic tissues. IL-17A target gene expression was undetectable in non-laminitic samples with the exception of weak detection of *DEFB4B*, *S100A9* and *PTSG2*. In contrast, all target genes examined, except *CCL20*, were expressed by some or all laminitic samples. By qPCR, severe acute (n = 7) SLL expressed ~15–100 fold higher levels of *DEFB4B* and *S100A9* genes compared to non-laminitic controls (n = 8). *DEFB4B* was also upregulated in developmental/subclinical (n = 8) and moderate acute (n = 7) by ~ 5-fold, and in severe chronic (n = 5) by ~15–200 fold. In situ hybridization (*DEFB4*) and immunofluorescence (calprotectin, a dimer of S100A9/S100A8 proteins) demonstrated expression in keratinocytes, primarily in suprabasal cell layers, from SLL samples. These data demonstrate upregulation of a cohort of IL-17A target genes in SLL and support the hypothesis that similarities in the response to stresses and damage exist between equine and human epidermal tissues.

## Introduction

**Fig 1:**
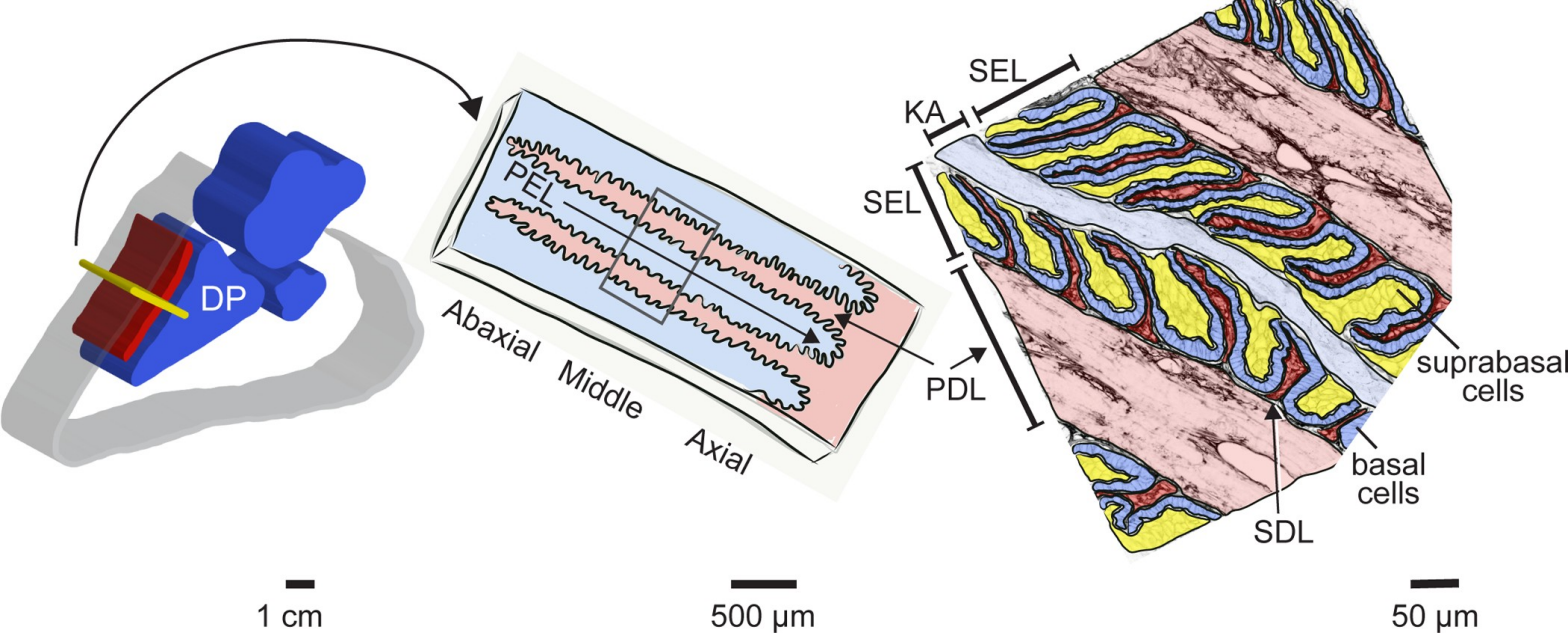
Schematic overview of equine hoof lamellar tissue anatomy. Left panel: cut away view of the midsagittal section of an equine foot outlining position of lamellar tissue (red) relative to hoof wall (grey) and distal phalanx (DP), middle phalanx, and distal sesamoid bones (blue). The transverse section plane used for tissue collection is shown in yellow. Center panel: a portion of the transverse section plane diagrammed schematically. The interdigitating primary epidermal lamellae (PEL) and primary dermal lamellae (PDL) are highlighted. Positions relative to the skeletal axis are denoted as axial (closest to DP), middle, and abaxial (farthest from DP and closest to the hoof wall). Right panel: higher magnification illustration of lamellar tissue organization. Colors are overlaid on a tissue section stained to highlight cell membranes and extracellular matrix (rhodamine-tagged wheat germ agglutinin, see Methods). Darker blue overlays mark basal epidermal cells, yellow overlays mark suprabasal epidermal cells, light blue overlay highlights the keratinized axis (KA) of each PEL. The PEL consists of combined secondary epidermal lamellae (SEL) and KA. Dermal tissues are shown in red overlays, with light red highlighting PDL and darker red marking the secondary dermal lamellae (SDL).

Equine laminitis is a common, progressive, crippling and currently incurable disease often necessitating euthanasia to end suffering. Healthy lamellar tissue connects the inner hoof wall to the distal phalanx (DP), forming a major component of the suspensory apparatus of the DP, a unique adaptation of equids that allows for suspension of almost the entire weight of the animal through the hoof capsule [1, 2]. The hoof is a modified epidermal appendage integrated into the musculoskeletal system by the epidermal and dermal lamellae, homologous to the nail bed, which in horses are extensively folded to form primary and secondary lamellae to increase the surface area of attachment (Fig 1; [3, 4]). Similar to skin, keratin 14 is expressed within lamellar epidermal basal cells [5–7]; however equine epidermal lamellae have several architectural and molecular modifications that differ from skin, including limited stratification into basal, suprabasal and cornified layers, and expression of unique keratins not found in equine or human skin [5–7]. These modifications are thought to impart critical functional differences that allow equine lamellar tissue to bear significantly greater mechanical tensile and compressive forces compared to skin, consistent with the functional integration of the hoof capsule and lamellae into the musculoskeletal system [8].

In laminitis, equine epidermal lamellae become elongated, distorted and detached, leading to loss of mechanical support and failure to suspend the DP within the hoof capsule and, with progression to chronic laminitis, transform into an aberrantly thickened and dysplastic structure termed the "lamellar wedge" [2, 9–13]. There are three broad categories of laminitis associated with different etiologic risk factors: supporting limb laminitis (SLL), resulting from increased weight-bearing on the affected limb after pain from injury or bacterial infection reduces weight-bearing on a contralateral limb; endocrinopathic laminitis (EL) in which hyperinsulinemia is the key risk factor; and sepsis-related laminitis (SRL), triggered by systemic inflammatory conditions (reviewed by [13]). While differences in etiopathogenesis are reported for the different types of laminitis, several shared morphologic and molecular similarities exist, including keratinocyte apoptosis/necrosis; epidermal hyperplasia, acanthosis, dyskeratosis and hyperkeratosis; basement membrane separation; dermal inflammation; and progressive, deforming distal phalangeal osteolysis with medullary stromal activation and inflammation [9, 11, 14–19].

**Fig 2:**
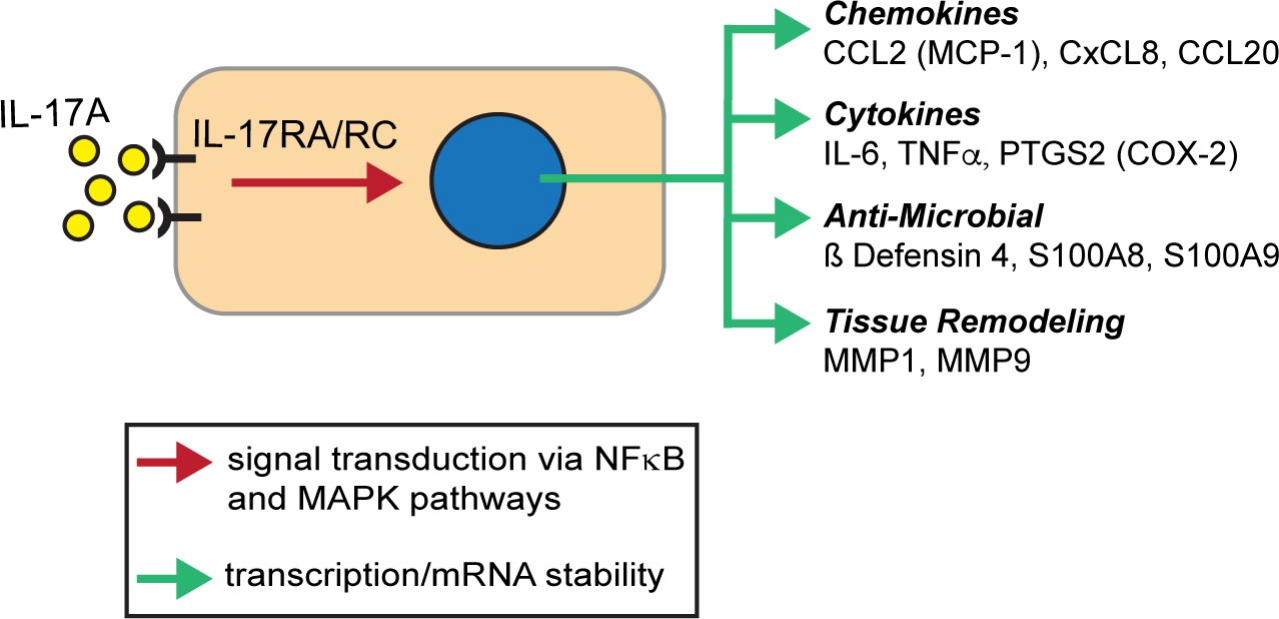
Overview of the IL-17A pathway and downstream target genes. IL-17A binding to receptor heterodimer of IL-17RA and RC subunits activates downstream pathways to activate either new gene expression or increased mRNA stability for a cohort of target genes. The 11 genes assayed in this study are shown and derived from the IL-17A signaling pathway in Homo sapiens [33]. See [21, 29, 34] for details on signal transduction.

Interestingly, several histomorphologic features of equine laminitis are similar to those described for both physiologic cutaneous wound-healing and select chronic auto-inflammatory skin conditions, including human psoriasis. Human psoriasis is a chronic, heterogeneous immune-mediated inflammatory disease with genetic and epigenetic triggers that activate pro-inflammatory pathways, including IL-17A [18, 20, 21]. A causal role for IL-17A-dependent inflammation in driving psoriasis is highlighted by the success of monoclonal antibody-based therapies that bind IL-17A and block signaling through the IL-17A receptor [18, 21, 22]. IL-17A stimulated inflammation is also associated with a number of other human diseases, including rheumatoid arthritis, obesity-associated inflammation and cancer [19, 21, 23–26], as well as physiologic cutaneous wound-healing. In human skin diseases and normal healing processes, both immune cells and keratinocytes respond to IL-17A directly via its receptor, a heterodimer of IL-17RA and IL-17RC (Fig 2; [18, 21, 27, 28]). Receptor activation results in increased expression of target genes either through enhanced translation or increased mRNA stability [21, 25, 29]. The cohort of target genes includes the ß defensins, with ß defensin 4 (human gene *DEF4B*) ranking as the most highly upregulated gene in human keratinocytes treated with IL-17A [30]. ß defensin 4 (also known as human ß defensin 2) is a biomarker for psoriasis and its decline provides a quantitative measure of immunotherapy efficacy [31, 32]. Additional effectors include cytokines, chemokines, antimicrobial proteins and matrix metalloproteinases [18, 21, 25, 29] as summarized in Fig 2.

Here we test the hypothesis that in SLL the equine lamellae respond to stress by expression of genes upregulated by IL-17A, similar to that found in human skin diseases or wound healing. Although natural cases provide a "temporal snap shot" of disease, typically at late or chronic stages where extensive damage has already occurred in the primarily affected foot, often horses euthanized for SLL demonstrate less severe stages of laminitis in other secondarily affected feet presumably due to related alterations in weight bearing [11]. These studies examined a cohort of archived lamellar tissues from Thoroughbred horses euthanized for spontaneously occurring SLL, including feet exhibiting varying stages and severity of disease. The expression of IL-17A, IL-17RA and eleven IL-17A target genes was examined by either RT-PCR or qPCR and compared to age and breed matched non-laminitic controls. Cellular localization was examined for two effectors by in situ hybridization (ß defensin 4; *E*. *caballus* gene name *DEFB4B*) or immunofluorescence (calprotectin, a dimer of S100A8/S100A9 proteins) to ask whether the keratinocytes themselves respond by upregulating expression of IL-17A target genes.

## Methods

### Ethics statement

The archived equine tissue samples used in this study were collected under protocols titled ‘Pathophysiology of Equine Laminitis (By-products only)’ and ‘Equine Laminitis Tissue Bank’. These protocols were approved by the University of Pennsylvania Institutional Animal Care and Use Committee (protocol #801950 and #804262, respectively). Euthanasia of the horses was carried out in accordance with the recommendations in the Guide for the Care and Use of Agricultural Animals in Research in Teaching Federation for Animal Science Societies) and the AVMA Guidelines for the Euthanasia of Animals (American Veterinary Medical Association) by overdose with pentobarbital sodium and phenytoin sodium.

### Subjects and tissue retrieval

Samples were obtained from the Laminitis Discovery Database, a tissue repository housed in the Galantino-Homer laboratory at the University of Pennsylvania School of Veterinary Medicine, New Bolton Center [35]. The cohort included 14 Thoroughbred SLL cases with clinical signs and gross and/or histological lesions consistent with laminitis in at least one limb and 7 age-matched Thoroughbred controls (Table 1). At the time of euthanasia, all control and SLL case Thoroughbreds were currently in, or recently retired due to injury, from racing or training to race, aged 2–7 years, and included females, males and castrated males, as listed in Table 1. All control horses and one SLL case were donated for research and teaching; the remaining SLL cases were client-owned animals submitted for necropsy following euthanasia due to SLL. Lamellar tissue, including epidermal and dermal lamellae, was collected immediately after euthanasia as previously described [7, 11, 35, 36]. Tissue samples were immediately either snap frozen in liquid nitrogen and stored in liquid nitrogen until processed for RNA extraction, or formalin-fixed/paraffin-embedded (FFPE) and sectioned for microscopy experiments [7, 36, 37]. Tissue sections were oriented transverse to the axis of the limb and include the entire abaxial, middle, and axial regions (relative to the DP) of the primary epidermal lamellae as well as the sub-lamellar dermal tissue located between the DP and epidermal lamellae (see Fig 1).

**Table 1:** Sample summary of signalment, duration of clinical signs of laminitis, gross pathology and histopathology for lamellar tissue from Thoroughbred horses used for RNA extraction.

SampleNumber	LDD case^a^	Age (yr)^b^	Sex^c^	SLL Foot^d^	Duration Score and (days)^e^	Gross Pathology Score^f^	Histopathology Score^g^	Laminitis Group^h^	Notes^i^
1	130 LF	4	MC	NA	0	1	1	Control	Donation, no history available
2	130 LH	4	MC	NA	0	1	1	Control	
3	127 RF	3	MC	NA	0	1	1	Control	Donation, no history available
4	127 RH	3	MC	NA	0	1	1	Control	
5	126 LF	3	F	NA	0	1	1	Control	Donation, no history available
6	126 LH	3	F	NA	0	1	1	Control	
7	51 LF	3	MC	NA	0	1	1	Control	Donation, Condylar fracture (limb not specified)
8	84 RF	2	MC	NA	0	1	1	Control	Donation, chronic orthopedic injury (not specified), scars on RF and RH fetlocks
9	148 RH	3	F	NA	0	1	1	Control	Donation, no history available
10	60 LF	6	MC	NA	0	1	1	Control	Donation, LF coffin bone deformity, not lame
11	128 LH	6	M	Ipsi. Hind	0	1	2	Develop-mental	RF (Contralateral): Severe chronic laminitis. LF catastrophic open, comminuted fracture of the medial proximal sesamoid bone (4 months); fetlock arthrodesis; post-operative surgical site infection and bacterial osteomyelitis of digit and metacarpus.
12	153 LF	7	MC	Injury	0	1	2	Develop-mental	RF (Contralateral): Moderate chronic laminitis. LF medial proximal sesamoid fracture (4 months), managed with pasture rest.
13	65 RH	4	F	Contra-lateral	0	1	2	Develop-mental	LH (injury): Complete transverse comminuted fracture of calcaneus (3 weeks) with sequestrum and septic tenosynovitis.
14	124 LH	6	M	Ipsi. Hind	0	1	2	Develop-mental	RF (Contralateral): Severe acute laminitis. LF catastrophic comminuted fractures of the medial and lateral proximal sesamoid bones with thrombosis of medial and lateral palmer metacarpal arteries and veins resulting in distal limb ischemia (5 days).
15	124 RH	6	M	Con. Hind	0	1	2	Develop-mental	
16	143 RH	3	M	Ipsi. Hind	0	1	2	Develop-mental	LF (Contralateral): Severe acute laminitis. RF catastrophic fracture of multiple carpal bones (14 days), carpal arthrodesis, post-operative septic jugular vein thrombophlebitis with secondary embolic pneumonia, nephritis and hepatitis.
17	153 RH	7	MC	Con. Hind	0	1	2	Develop-mental	see sample 12
18	164 LF	6	F	Injury	0	1	2	Develop-mental	RF (Contralateral): Severe chronic laminitis. LF catastrophic injury (3 weeks); fetlock arthrodesis; post-operative surgery site infection.
19	118 RF	2	F	Injury	0	2	2	Moderate Acute	RF (Injury): catastrophic comminuted fractures of the medial and lateral proximal sesamoid bones (1 month); fetlock arthrodesis; post-operative subluxation of proximal interphalangeal joint.
20	128 RH	6	M	Con. Hind	0	2	2	Moderate Acute	RF (Contralateral): Severe chronic laminitis. See note for 128 LH.
21	163 LH	3	F	Ipsi. Hind	1 (<1)	2	2	Moderate Acute	RF (Contralateral): Severe chronic laminitis. LF medial quarter crack and chronic hoof abscessation (at least 6 weeks).
22	106 RF	2	F	Injury	2 (2)	3	2	Moderate Acute	LF (Contralateral): Severe acute laminitis. RF: Septic middle carpal joint (5 weeks).
23	143 LH	3	M	Con. Hind	2 (2)	2	2	Moderate Acute	LF (Contralateral): Severe acute laminitis. See note for 143 RH (sample #16).
24	155 LF	3	M	Contra-lateral	2 (7)	2	2	Moderate Acute	LF (Contralateral): Acute laminitis. RF comminuted fracture of radial carpal bone (90 days) managed with stall rest.
25	155 RF	3	M	Injury	2 (7)	2	2	Moderate Acute	
26	99 LF	2	F	Contra-lateral	2 (7)	4	3	Severe Acute	LF (Contralateral): Severe acute laminitis. RF condylar fracture (2 weeks) with surgical repair; post-operative injury of RF elbow and shoulder associated with stall confinement.
27	99 RF	2	F	Injury	2 (3)	4	3	Severe Acute	
28	106 LF	2	F	Contra-lateral	2 (7)	4	3	Severe Acute	LF (Contralateral): Severe acute laminitis. See note for 106 RF (sample #22).
29	115 RF	6	F	Injury	2 (5)	4	3	Severe Acute	LF (Contralateral): Severe acute laminitis. RF catastrophic injury (24 days); fetlock arthrodesis; no evidence of infection.
30	118 LF	2	F	Contra-lateral	2 (5)	4	3	Severe Acute	LF (Contralateral): Severe acute laminitis. See note for 118 RF (sample #19).
31	124 RF	6	M	Contra-lateral	2 (2)	4	3	Severe Acute	RF (Contralateral): Severe acute laminitis. See note for 124 LH (sample #14).
32	143 LF	3	M	Contra-lateral	2 (4)	4	3	Severe Acute	LF (Contralateral): Severe acute laminitis. See note for 143 RH (Sample #16).
33	128 RF	6	M	Contra-lateral	3 (60)	4	3	Severe Chronic	RF (Contralateral): Severe chronic laminitis. See note for 128 LH (sample #11).
34	163 LF	3	F	Injury	3 (>30)	4	3	Severe Chronic	LF (Injury) and RF (Contralateral): Severe chronic laminitis. See note for 163 LH (sample #21)
35	163 RF	3	F	Contra-lateral	3 (>30)	4	3	Severe Chronic	
36	40 RF	5	M	Con. Front	3 (60)	4	3	Severe Chronic	RH (Contralateral): Severe chronic laminitis. LH (Injury): Chronic necrotizing bacterial cellulitis of the hock with suspect septic arthritis and tenosynovitis (2 months)
37	66 LF	6	F	Contra-lateral	3 (12–20)	4	3	Severe Chronic	LF (Contralateral): Severe chronic laminitis. RF (Injury): Catastrophic comminuted fractures of the proximal sesamoid bones with disruption of superficial and deep digital flexor tendons (3 weeks); fetlock arthrodesis; post-operative orthopedic implant infection and distal digital ischemic necrosis
38	164 RF	6	F	Contra-lateral	3 (7–14)	4	3	Severe Chronic	RF(Contralateral): Severe chronic laminitis. See note for 164 LF (sample #18).

^a^Sample identification is listed by Laminitis Discovery Database horse identification number and foot (LF: Left Front; RF: Right Front; LH: Left Hind; RH: Right Hind).

^b^Age given in years.

^c^Sex (F: Female; M: Male; MC: Castrated Male)

^d^SLL Foot identified relative to the primary injury in horses with Supporting Limb Laminitis (SLL). NA: Not Applicable (Control); Injury: foot or limb with primary injury; Contralateral: Foot contralateral to injury limb; Ipsi. Hind: Hind foot ipsilateral to primary injury limb; Con. Hind: Hind foot contralateral to primary injury limb.

^e^Duration Score indicates the duration of the history or clinical signs of laminitis as 0: None, 1: one day or less (early acute), 2: 2–7 days (acute), or 3: 7–30 days (chronic). Duration in days provided parenthetically after each score.

^f^Gross Pathology Score listed as 1: Normal/minimal, 2: Mild, 3: Moderate, or 4: Severe, based on presence of lesions consistent with laminitis, as previously described [38].

^g^Histopathology Score listed as 1: Normal/Mild, 2: Moderate, or 3: Severe, based on the presence of lesions consistent with laminitis, as previously described [38].

^h^Laminitis Group indicates how samples were grouped for analysis of the data as described in the Methods section and Table 2.

^i^Information is provided on the severity and duration of lesions consistent with laminitis detected in additional feet from the same horse. Available information regarding the original injury is also provided with known duration of injury provided parenthetically. Control horses were donated for teaching or research with minimal historical information.

SLL cases were defined by the presence of a primary orthopedic injury with or without infection that resulted in aberrant weight bearing in the affected limb, as reported by the attending veterinarian, with clinical expression of signs compatible with laminitis in at least one other limb at the time of euthanasia, and confirmed by gross pathology and histopathology evaluations as described below. The 7 age-matched controls were euthanized primarily for lameness due to non-laminitic orthopedic disease and showed few gross or histological lesions compatible with laminitis (Table 1).

### Antemortem clinical characterization and postmortem gross/histological evaluation

Each foot was scored for antemortem presence and duration (in days) of clinical signs compatible with laminitis, as well as gross and histologic postmortem lesions compatible with non-laminitis controls and 4 SLL categories (developmental/subclinical, moderate acute, severe acute, or severe chronic), as follows and as summarized in Table 2: **Non-laminitic**: No clinical signs of laminitis, a normal or minimal gross pathology score (gross pathology score 1) and no or minimal histological lesions compatible with laminitis (histopathology score 1); **Developmental/subclinical laminitis**: No clinical signs of laminitis, a normal or minimal gross pathology score (gross pathology score 1), but histological lesions compatible with moderate lamellar damage are present (histopathology score 2); **Moderate acute laminitis**: 0–7 days duration of active clinical signs of laminitis, moderate gross pathology lesions (gross pathology score of 2–3), and histological lesions compatible with moderate laminitis (histopathology score 2). Two samples (samples #19, #20, Table 1) had no reported clinical signs of laminitis (duration score: 0), but were included in the Moderate Acute group because both had gross lesions consistent with laminitis (score: 2) in contrast to the Developmental/subclinical group, none of which had gross lesions consistent with laminitis (score: 1). It was assumed that clinical signs of moderate laminitis in those two feet were masked or missed due to more severe disease in other limbs and/or the presence of analgesic therapeutics; **Severe acute laminitis**: 1–7 days duration of active clinical signs of laminitis, severe gross pathology lesions (gross pathology score of 4), and histological lesions compatible with severe laminitis (histopathology score 3); **Severe chronic laminitis**: > 7 days duration of active clinical signs of laminitis, severe gross pathology lesions (gross pathology score of 4), and histological lesions compatible with severe laminitis (histopathology score 3).

**Table 2:** Laminitis group parameters.

Laminitis Group	Clinical Duration Score^a^	Gross Pathology Score^b^	Histopathology Score^c^
Control	0	1	1
Developmental/subclinical	0	1	2
Moderate Acute	0–2	2–3	2
Severe Acute	2	4	3
Severe Chronic	3	4	3

^a^Duration Score indicates the duration of the history or clinical signs of laminitis as 0: None, 1: one day or less (early acute), 2: 2–7 days (acute), or 3: 7–30 days (chronic). Two samples with no reported clinical signs (duration score: 0) were included in the Moderate Acute category due to the presence of gross lesions consistent with laminitis (score: 2) as detailed in the Methods section.

^b^Gross Pathology Score listed as 1: Normal/minimal, 2: Mild, 3: Moderate, or 4: Severe, based on presence of lesions consistent with laminitis, as previously described [38].

^c^Histopathology Score listed as 1: Normal/Mild, 2: Moderate, or 3: Severe, based on the presence of lesions consistent with laminitis, as previously described [38].

Gross pathology was assessed from direct observations and photographic images captured immediately after mid-sagittal sectioning of the foot and was scored to group SLL limbs into 4 severity categories (1: minimal or no lesions; 2: mild lesions; 3: moderate lesions; 4 severe lesions) based on the presence of lesions as previously described [38]). Quantitative and qualitative histopathology was assessed on FFPE mid-dorsal lamellar tissue samples that were sectioned and stained with hematoxylin and eosin stain and with Periodic acid-Schiff (PAS)-hematoxylin (PASH) stain, as described previously [38]. The detailed histopathology scoring and analysis for the sample set used for this study will be published separately. The summary histopathology scores presented in Tables 1 and 2 represent: 1) Normal/Mild, none or focal distribution of lesions compatible with laminitis, 2) Moderate, multifocal to regional distribution of lesions compatible with laminitis, or 3) Severe, global distribution of lesions compatible with laminitis. The evaluation of histopathology lesions was performed as previously described [38].

### Oligonucleotide primers

Primers were designed to amplify equine sequences encoding regions of IL-17RA (receptor subunit A), IL-17A, and 11 products of the IL-17A pathway (Table 3). Primer sequences for several of the target genes were based on previous publications (Table 3). Additional primers were designed using tools available through either NCBI [39] or OligoPerfect Primer Designer tool [40]. *RACK1* (formerly named *GNB2L1*) was used as the reference gene for PCR-based experiments based on previous work demonstrating stable expression of this gene in lamellar tissue from horses with experimental laminitis and non-laminitic controls [41]. The *RACK1* primers amplify a region spanning an intron/exon border and amplify a 200 bp sequence from cDNA and a 741 bp sequence from genomic DNA, providing confirmation that all cDNA samples were free of genomic DNA contamination. With the exception of *IL6* and *PTGS2*, primers for target genes also spanned more than one exon, also confirming that experiments identified fully processed mRNAs. Oligonucleotide primers were synthesized by Integrated DNA Technologies, Inc. (Coralville, IA, USA).

**Table 3:** Equine PCR primer sequences.

Gene Name	Common Name	NCBI Accession Number	Product Size (bp)	Forward primer (5' - 3') Reverse primer (5' - 3')	T_m_ (°C)	Reference or design tool
*CCL2*	CCL2, MCP-1	NM_001081931.2	286	TCTCCAGTCACCTGCTGCTA ATATCAGGGGGCATTTAGGG	55	[40]
*CCL20*		XM_003365131.4	296	CAGCAAGTCAGAAGCAGCAAGC AGCAACTCCAGCCAGGTTCT	59	[39]
*CxCL8*	CxCL8, IL-8	NM_001083951.2	297	CAGGGACAGCAGAGACACAA GCTCCGTTGACGAGCTTTAC	57	[39]
*DEFB4B* (primer pair 1)	ß defensin 4B	NM_001081887.1	149	ATTTTCTACTTGCCTTCCTCAT GATACAAGTGCCGATCTGT	52	[42]
*DEFB4B* (primer pair 2)	ß defensin 4B	NM_001081887.1	105	GTCTTCCTGTTGCCTGTTCCA TGACCCTGGAAGGCACTTAG	57	[39]
*IL6*	IL-6	NM_001082496.2	152	CACCGTCACTCCAGTTGCCTT GGATGTACTTAATGTGCTGTTTGGTTTTGTCTG	59	[43]
*IL17A*		NM_001143792.1	146	TGAGTCTGGTGGCTATCGTG ATCTGAGGCCCTTCTGGAAT	55	[39]
*IL17RA*	IL-17 Receptor A	XM_005610824.3	254	ATATGCCGCTGTGGGTGTA TGGGCAAACTTTAGGACCAC	55	[40]
*MMP1*	MMP-1	NM_001081847.2	174	CAGTGCCTTCAGAAACACGA GCTTCCCAGTCACTTTCAGC	55	[40]
*MMP9*	MMP-9	NM_001111302.1	188	TCGACGACGAAGAGTTGTGG GCGGTCGGTGTCATAGTAGG	57	[39]
*PTGS2*	COX-2	NM_001081775.2	203	GTATCCGCCCACAGTCAAAGA ACAAGCGTTCATCATCCCATTC	57	[43]
*RACK1*	Receptor for Activated C Kinase 1. formerly GNB2L1	NM_001242446.1	200	CAGGGATGAGACCAACTACG ATGCCACACTCAGCACATC	55	[41]
*S100A8*	S100 calcium binding protein A8	XM_001493589.4	140	ATTCTGTTTCGGGGAGACCT CGTCCCTATAGATGGCATGG	55	[40]
*S100A9*	S100 calcium binding protein A9	XM_001494378.5	142	GCTCAAGGTTACCACACTGC TTTCTGCACTCCAAGCCGAG	57	[39]
*TNF*	TNFα	NM_001081819.2	219	AGCCCATGTTGTAGCAAACC GAGACAGCTAAGCGGCTGAT	57	[40]

Gene names are as given for the NCBI accession numbers. Melting temperatures (T_m_) were provided by Integrated DNA Technologies.

### RNA extraction and cDNA synthesis

Archived snap-frozen tissue was pulverized and RNA extracted as described in detail previously [7]. Briefly, total RNA was extracted from pulverized tissue using the RNeasy Fibrous Tissue Mini Kit (Qiagen, Valencia, CA, USA) with modifications from the manufacturer’s instructions necessary for the highly fibrous lamellar tissue. Pulverized tissue samples were vortexed gently in 900 μl of Buffer RLT and proteinase K, incubated at 55°C for 10 min and homogenized by passing 5-10x through an 18 g hypodermic needle and syringe. The resulting material was clarified at 10,000 x g for 10 min. The supernatant was combined with 0.5 volume of 100% ethanol and the solution applied to the RNeasy Mini column in 700 μl increments, and then spun at 10,000 x g. RNA was eluted from the RNeasy column by adding 350 μl Buffer RW1 and centrifugation for 15 s at 10,000 x g. DNase treatment and subsequent buffer RW1 and RPE steps were then performed according to the manufacturer’s instructions. RNA was eluted in a total volume of 50 μl of RNase-free water. Total RNA was quantified using a Nanodrop 2000 spectrophotometer (Thermo Fisher Scientific, Waltham, MA, USA). The quality of the RNA preparations was confirmed by A260/A280 ratios of 2.0 ± 0.1 for all samples used here.

cDNAs were prepared from each RNA sample using a TaqMan^TM^ reverse transcription kit using random hexamer primers (ThermoFisher Scientific, catalog number N8080234) according the the manufacturer's instructions. Total reaction volumes were 20–40 μl and included 100 ng of RNA per each 20 μL volume. Reverse transcription was carried out for 30 min at 37°C.

### RT-PCR

PCR amplification reactions of 25 μL total volume included ThermoPol reaction buffer, 10 ng cDNA template, 0.625 units Taq DNA polymerase (New England Biolabs), 0.2 μM forward and reverse primers (Table 3), and 200 μM dNTPs. Amplification reactions were run using an Eppendorf Masterflex thermocycler. Amplimers were detected by electrophoresis on 2% agarose (Sigma-Aldrich) gels and visualized with ethidium bromide (MP Biomedicals LLC., Irvine, CA). Gels were imaged using a BioRad ChemiDoc MP system running Image Lab 5.1 software and exported as TIFF files. The black/white scale was inverted in Photoshop (version CC 2018; Adobe) for clarity.

### qPCR

The qPCR amplification reactions totaled 20 μL that included Power SYBR Green PCR Master Mix (Applied Biosystems), 0.5 μM forward and reverse primers, and 5 ng template cDNA. Amplifications were conducted using an Applied Biosystems 7300 Real Time PCR system and sequence detection software (version 1.4). All samples for qPCR were run in triplicate. The relative quantitation software package was used to determine fold changes in expression for *DEFB4B* and *S100A9*. The software uses the comparative C_T_ (ΔΔ C_T_) method. All fold changes reported here are based on *RACK1* as the reference gene for normalization, and are relative to expression of the target gene in a single non-laminitic control case (sample 1; Table 1). The qPCR analyses were limited to *DEFB4B* and *S100A9* because the other target genes were expressed at undetectable levels in the non-laminitic controls. Two primers were used to confirm the expression of *DEFB4B* (Table 3).

The fold-change values within each group were not normally distributed and therefore data were compared using non-parametric tests. Kruskal-Wallis rank sum test was used to compare all groups, followed by post hoc Wilcoxon-Mann-Whitney rank sum tests to compare all pairwise combinations. The analyses were performed using Kaleidagraph software (version 4.5.2).

### In situ hybridization

The entire 345 bp mRNA sequence of *DEFB4B* (NM_001081887.1) was used to synthesize a corresponding cDNA sequence. To facilitate cloning, 5’ NotI and 3’ XhoI restriction sites were included in the synthesized DNA fragment. The synthesized DNA was cloned into pBluescript SK (+) vectors at the multi-cloning site, which includes T7 and T3 RNA polymerase promoter sequences. Gene synthesis, cloning, and verification of sequence were performed by Genscript (genscript.com). Digoxigenin (DIG)-labeled riboprobes were synthesized from T7 (for antisense probe synthesis) and T3 (for sense probe synthesis) promoters using MEGAscript Transcription kits (Ambion, Thermo Fisher Scientific) according to the manufacturer’s protocol.

In situ hybridization was performed using standard methods [44] as described previously [7], modified to include antibody incubation at room temperature. Briefly, tissue sections were deparaffinized in xylene, followed by rehydration in a graded ethanol series (100%, 75%, 50% and 25%) and digested for 5 min with proteinase K (10μg/mL; Ambion). Tissue sections were allowed to hybridize overnight in a humid chamber at 65°C with 1 ng/μL of sense (negative probe) or antisense (positive probe) DIG-labeled riboprobes in hybridization buffer containing 50% formamide. After washes in saline-sodium citrate buffer, the sections were incubated with alkaline phosphatase-conjugated anti-DIG Fab fragments (#11093274910, 1:3000, SigmaAldrich, St. Louis, MO, USA) in a humid chamber for 1 h at 37°C. After washing in PTB (Phosphate Buffered Saline + 0.2% Triton x-100 + 0.1% BSA), labeled probe was visualized using NBT/BCIP substrate (Roche Diagnostics, Indianapolis, IN, USA) resulting in a blue/purple precipitate. PTw buffer (Phosphate Buffered Saline + 0.1% Tween) was used to stop the reaction. Sections were mounted in 80% glycerol/PTw.

Sections from PASH or in situ hybridization were imaged using a Nikon Nti microscope, a Nikon DS /Ti2 color camera and Nikon Elements software (Nikon Instruments, Inc., Melville, NY, USA). Images were collected using 4X or 10X objectives. Images collected at 4X were manually stitched together using Adobe Illustrator, allowing visualization of entire sections.

### Immunofluorescence

Tissue sections were also used to localize calprotectin using mouse antibody ab22506 (1:5000; Abcam) recognizing S100A9, followed by goat anti mouse IgG-Alexa 488 (1:100), based on a protocol described previously [38]. The calprotectin antibody has been used previously in equine tissues [16, 45, 46]. To confirm identity of keratinocytes, mouse monoclonal anti-keratin 14 antibody (Novus Biologicals; LL02, 1:500) was used in place of the anti-calprotectin antibody [47]. Rhodamine-tagged wheat germ agglutinin (12.5 μg/ml; Vector Laboratories) was used as a counterstain to outline cell membranes and extracellular matrix, facilitating identification of lamellar microanatomy [37]. Sections were imaged using a Zeiss 880 confocal microscope system including a Zeiss Axio Observer inverted microscope stand and 25X PlanApo objective (0.8 NA), controlled by Zen software (version 2.1). Large areas of tissue were imaged using the tile scan feature, typically set to 0% overlap between sections. The tiled sections were stitched together using Zen software (version 2.1).

## Results

### IL-17 receptor subunit A and IL-17A expression

**Fig 3:**
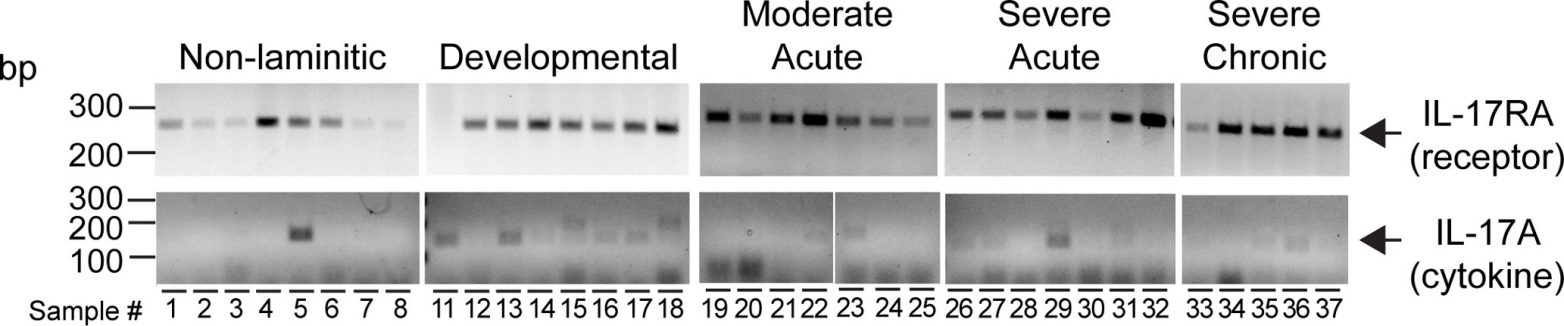
*IL-17A* and its receptor, *IL17-RA* are expressed in equine lamellar tissue. RT-PCR products amplified from equine lamellar tissue isolated from non-laminitic controls and the 4 SLL disease states are shown as indicated. Developmental refers to the developmental/subclinical group. Base pair lengths are indicated to the left of gels. Most samples expressed detectable levels of *IL-17RA*. Expression of *IL-17A* was weak, but more frequently detected in laminitic tissue samples. Sample numbers are listed in Table 1.

Before examining downstream effectors of the IL-17A pathway, we first determined that *IL-17RA*, encoding one half of the IL-17A receptor heterodimer, was detectable in most of the samples examined (Fig 3). The receptor subunit was detected in both non-laminitic and laminitic samples. Expression of *IL-17A* by RT-PCR was also detected, primarily in laminitic tissues, but also detected in one of the non-laminitic controls (Fig 3). To examine whether lamellar tissue responds to IL-17A, we next examined target gene expression.

### *DEFB4B* expression

**Fig 4:**
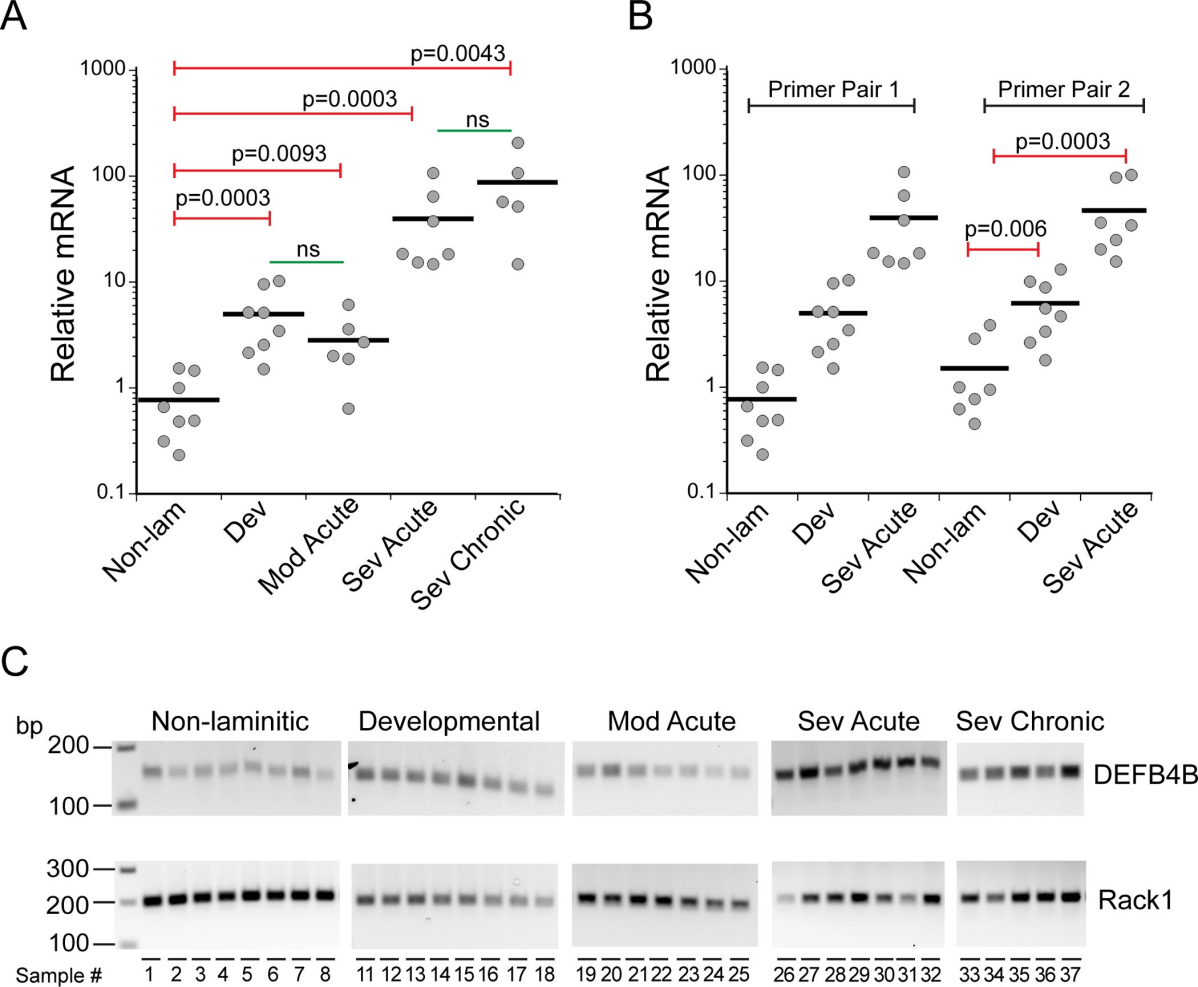
Equine *DEFB4B* expression was upregulated in SLL. (A,B) Relative *DEFB4B* gene expression measured by qPCR (see Methods) in SLL disease states (developmental/subclinical (Dev), moderate acute (Mod Acute), severe acute (Sev Acute), and severe chronic (Sev Chronic)) compared to non-laminitic controls (Non Lam) as indicated. (A) Relative mRNA levels are shown from qPCR using primer pair 1. (B) Upregulation of *DEFB4B* was verified with primer pair 2. See S1 Table for fold change values for each sample. Primer sequences are listed in Table 3. The p values are derived from Kruskal-Wallis and Wilcoxon-Mann-Whitney rank sum tests. From pairwise comparison of all disease groups, *DEFB4B* upregulation was not statistically significant between the two less severe disease groups or between the more severe groups (labeled ns). All pairwise comparisons are given in S2 Table. (C) End points from qPCR show amplimers of the predicted 149 bp for primer pair 1. Base pair lengths are indicated to the left of gels and sample numbers are indicated below each lane. The reference gene, *RACK1*, is also shown for the cohort of tissue samples examined. Sample information is listed in Table 1.

Given the high expression of ß defensin 4 (human gene name *DEFB4*) in IL-17A-treated human keratinocytes [30], we used qPCR to examine relative expression levels of *DEFB4B* mRNA in SLL samples compared to non-laminitic controls (Fig 4). Note that the equine *DEFB4B* sequence was formerly identified as ß defensin 1 [42]. As shown in Fig 4A, *DEFB4B* expression was upregulated at all disease stages compared to the non-laminitic controls. Modest (~5-fold) upregulation was detected in samples from developmental/subclinical and moderate acute stages. More robust (~15–100 fold) upregulation was detected in either severe acute or severe chronic samples. These results were confirmed using a second primer pair (Table 3) for both developmental/subclinical and severe acute samples (Fig 4B). End points from the qPCR experiment (primer pair 1) are shown in Fig 4C, confirming that primers amplified a sequence of the expected size (Table 3).

### Expression of other effectors of IL-17A-dependent signals

**Fig 5:**
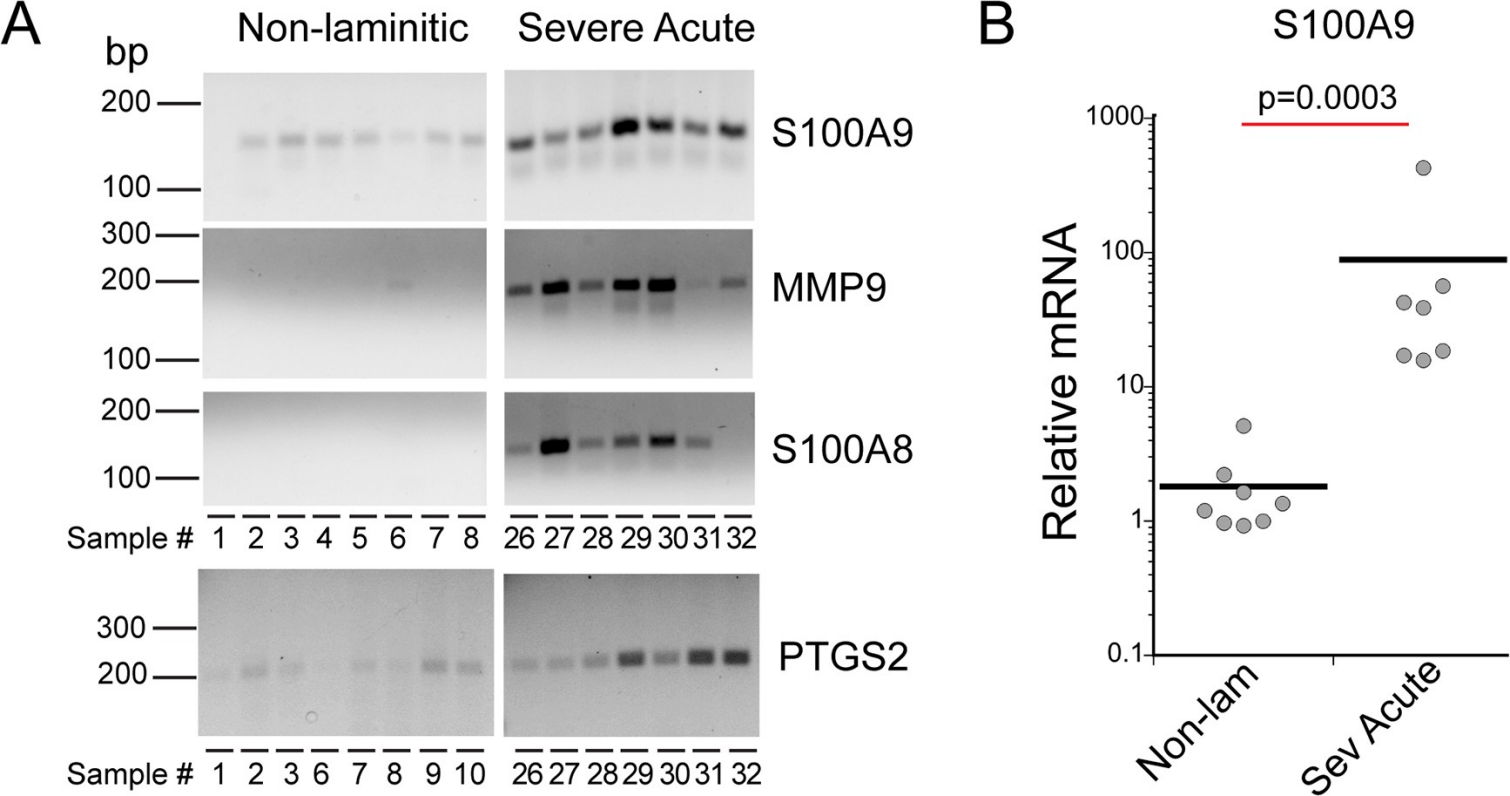
Four IL-17A target genes were expressed in most cases of severe acute SLL. (A) Amplimers are from either the qPCR end points (*S100A9*, *RACK1*) or from RT-PCR amplification, using the primer sequences listed in Table 3. *RACK1* serves as the reference gene for qPCR. Base pair lengths are indicated to the left of gels and sample numbers are indicated below each lane. Sample information is listed in Table 1. (B) *S100A9* was significantly upregulated in severe acute SLL. The p value was calculated by Wilcoxon-Mann-Whitney rank sum test. S1 Table lists fold changes for each sample, S2 Table includes p value for Wilcoxon-Mann-Whitney rank sum test.

As shown in Fig 5, *S100A9*, *MMP9*, *S100A8*, and *PTGS2* (common name, COX2, see Table 3) were expressed in severe acute SLL samples, while nearly undetected in the non-laminitic samples. For *S100A9*, the severe acute SLL samples showed significant upregulation of gene expression by qPCR (Fig 5B). The low or undetected expression in the non-laminitic samples for *MMP9*, *S100A8* or *PTGS2* made it impossible to calculate a fold change for these genes, but they were all clearly expressed in most, or all, of the severe acute samples examined. All amplimers were detected at the correct size (Table 3).

**Fig 6:**
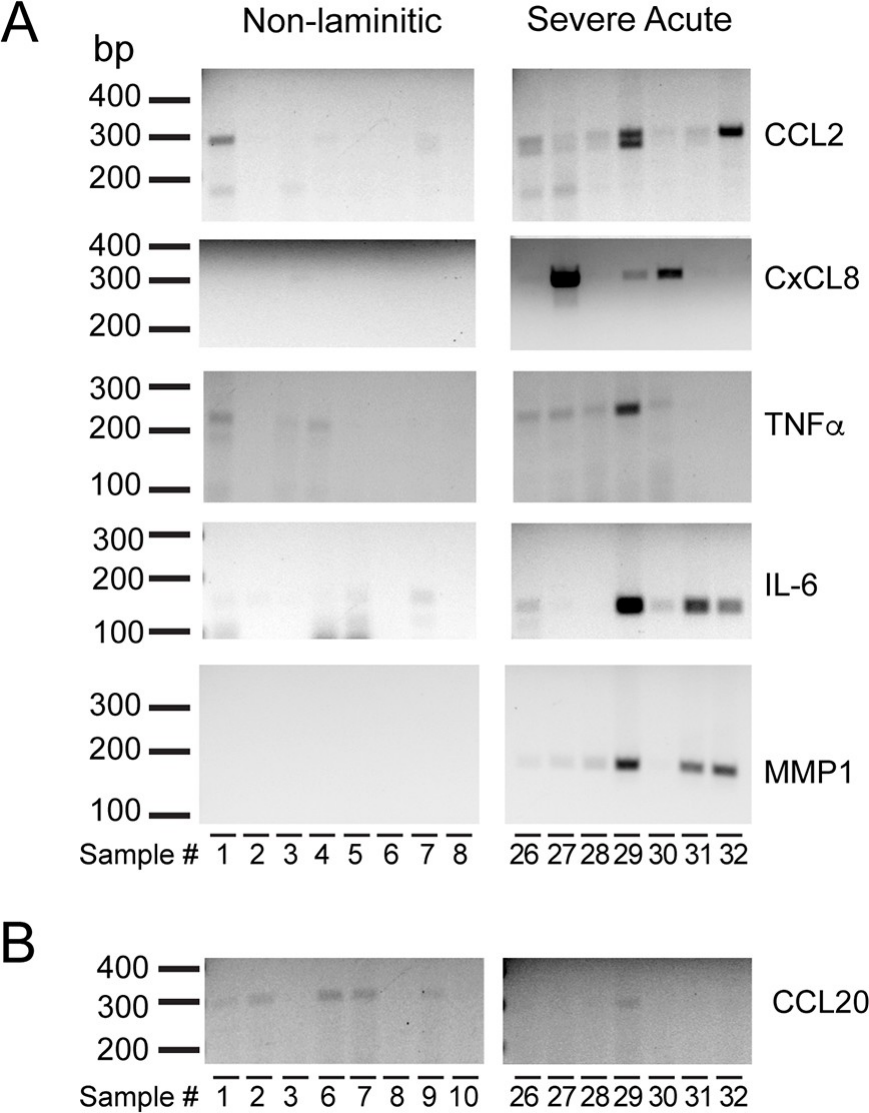
The expression of five IL-17A target genes was detected in some, but not all, severe acute SLL cases. (A) Amplimers from RT-PCR for *CCL2*, *CxCL8*, *TNFα*, *IL6*, and *MMP1* are shown for both non-laminitic controls and severe acute SLL. (B) *CCL20* was not detected in severe acute SLL. Base pair lengths are indicated to the left of gels and sample numbers are indicated below each lane. Sample information is listed in Table 1.

Expression of additional IL-17A target genes in severe acute SLL are shown in Fig 6A for *CCL2*, *CxCL8*, *TNFα*, *IL6* and *MMP1*. In the non-laminitic controls, gene expression was undetected, or very weakly detected in all cases. For the severe acute cases, gene expression was detected in some, but not all cases examined. An additional chemokine, *CCL20*, was weakly detected in non-laminitic controls and not detected in severe acute disease samples (Fig 6B).

Gene expression was also examined in samples from the other disease stages. Several of the IL-17A target genes were also detected in some, but not all, samples from developmental/subclinical and moderate acute disease stages (S1 Fig). Most of the developmental/subclinical samples expressed *PTGS2* and *IL6*, with fewer samples expressing detectable amplimer bands for *CCL2* or *TNFα*. The moderate acute samples showed detectable expression of *PTGS2*, *CCL2*, *TNFα*, and *IL6*. For the severe chronic cases, *CCL2*, *CxCL8*, *IL6*, *TNFα*, *S100A8*, *S100A9*, *MMP1* and *MMP9* were examined and these were expressed in the majority of severe chronic cases (S1 Fig).

### Localization of cells expressing *DEFB4B* and calprotectin in SLL

**Fig 7:**
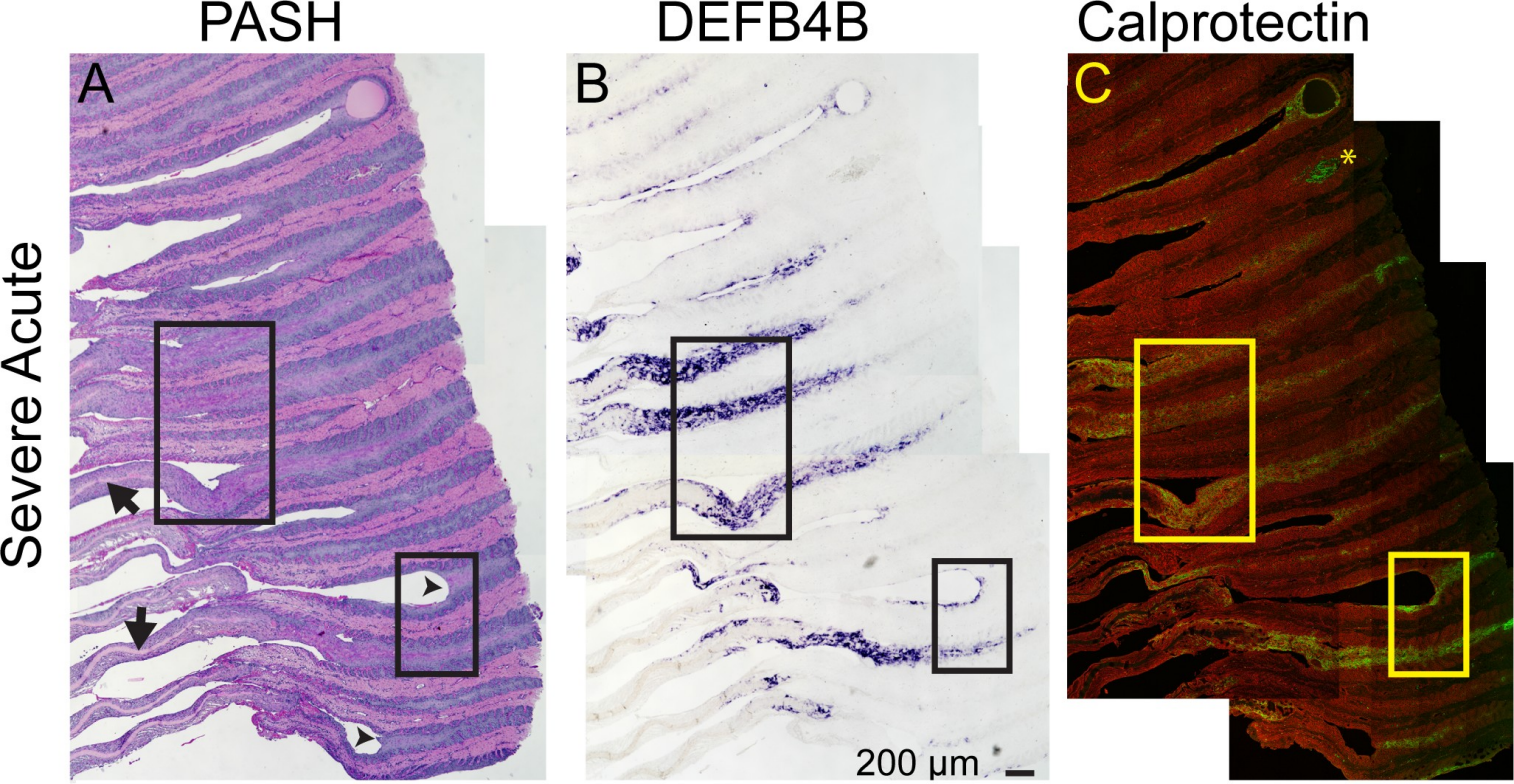
*BDEF4B* and calprotectin were expressed by lamellar keratinocytes in severe acute SLL. Images shown are from sequential FFPE lamellar tissue sections labeled by PASH staining, *DEFB4B* in situ hybridization to localize mRNA (purple color), and calprotectin (antibody recognizing S100A9 protein; green color) by immunofluorescence (n = 3 using samples 27, 32, and 38; images from sample 32 shown, see S2 Fig for images from sample 27 (severe acute) and 38 (severe chronic). Arrows in the PASH image mark locations of the centralized keratinized axis (KA) of the PEL, which have been displaced or "stripped" axially, from the axial remnants of the epidermal lamellae (arrowheads). Asterisk in the calprotectin image marks red blood cell autofluorescence. The calprotectin image also includes rhodamine-tagged wheat germ agglutinin (red) as a counterstain to mark both extracellular matrix and cell membranes. Each panel is a composite of multiple images tiled together (see Methods). The two boxed regions are shown at higher magnification in Fig 8. Sections are oriented with the abaxial region to the left and the axial region to the right.

**Fig 8:**
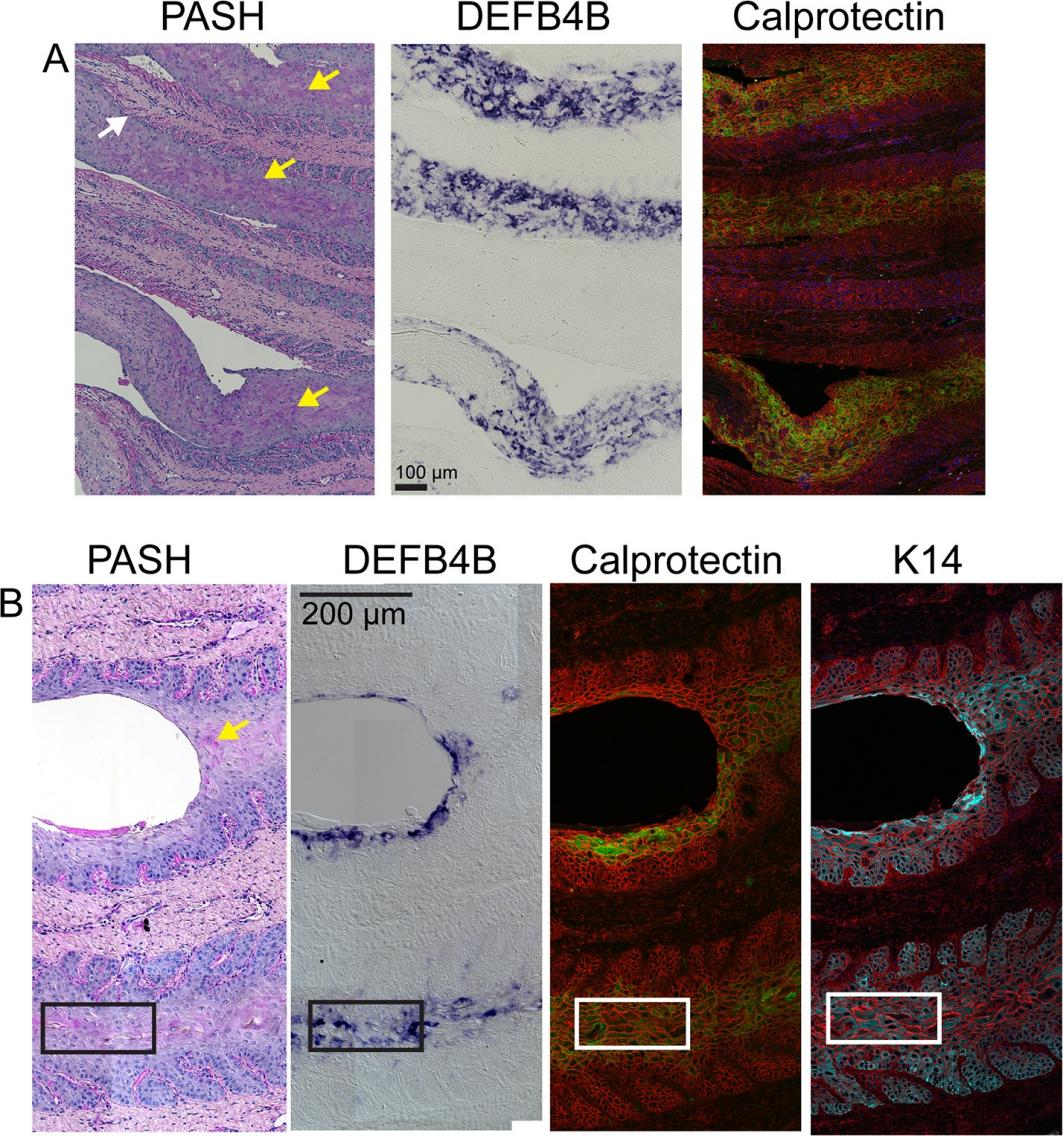
*DEFB4B* and calprotectin localize to suprabasal keratinocytes in areas that also demonstrate acanthosis and aberrant PAS-positive staining. Regions boxed in Fig 7 are shown at higher magnification for a severe acute SLL sample. *DEFB4B* mRNA was localized by in situ hybridization (purple color product) and calprotein protein by immunofluorescence (green). The calprotectin image also includes rhodamine-tagged wheat germ agglutinin (red) as a counterstain to mark both extracellular matrix and cell membranes. Regions of PAS positive (purple) keratinocytes are marked by yellow arrows. PAS also highlights the basement membrane separating dermal and epidermal layers (white arrow). Panel A corresponds to the upper box in Fig 7, Panel B corresponds to the lower box. Panel B includes differential interference in the *DEFB4B* image, and an additional sequential section showing localization of (keratin 14) K14 (cyan; also counterstained with rhodamine-tagged wheat germ agglutinin) to show the identification of these cells as keratinocytes. Note that K14 is typically expressed in basal cells but is also present in suprabasal cells in SLL [47].

To address whether IL-17A target genes are expressed by keratinocytes of the epidermal lamellae, we examined expression of two IL-17A target genes. *DEFB4B* expression was localized by in situ hybridization to detect the mRNA, and calprotectin was localized by immunofluorescence using a commercial antibody recognizing S100A9. Sequential sections were used for the two localizations and compared to PASH stained sections. Fig 7 shows tissue sections from a severe acute case stained by PASH (Fig 7A), and corresponding sections to localize *DEFB4B* expression (Fig 7B) and calprotectin (Fig 7C). The two boxed regions are shown at higher magnification in Fig 8. For both regions, *DEFB4B*- and calprotectin-expressing cells colocalize to the same areas, primarily suprabasal keratinocytes of the epidermal lamellae. These areas also include cells that aberrantly stain positively with PAS (arrows in Fig 8). Additional images from a second severe acute case and a severe chronic case are shown in S2 Fig; these also demonstrate co-localization of PAS-positive cells, *DEFB4B* and calprotectin to the same areas of the tissue and are detected primarily in suprabasal keratinocytes. The location of *DEFB4B* and/or calprotectin positive keratinocytes varied between individual cases and ranged from cells found in abaxial regions (S2 Fig), axial regions (Fig 7), or in regions adjacent to gross tissue damage and loss of tissue integrity (Figs 7 and 8).

Several controls confirmed the specificity of localizations for *DEFB4B* mRNA or calprotectin. First, neither *DEFB4B* nor calprotectin were detected in sections from non-laminitic tissues (S3 Fig). Second, a sense probe generated from the *DEFB4B* sequence did not bind tissue sections other than a weak binding to some cell nuclei (S4 Fig). Suprabasal cells within normal epidermal lamellae do not stain positively with PAS and have extremely limited stratification (S3 Fig).

## Discussion

We found significant evidence for upregulation of the IL-17A pathway in lamellar tissue isolated from horses euthanized for SLL based on the detection and upregulation of 10 out of 11 IL-17A target genes examined (Figs 4–6). For most IL-17A target genes, expression was undetectable in the non-laminitic samples, and therefore it was not feasible to calculate a gene expression fold change in the SLL samples. Two genes, *DEFB4B* and *S100A9*, were detected at sufficient levels in the non-laminitic tissue for relative quantitation. Both genes were expressed at ~ 15–100 fold higher levels in tissue from the severe acute stage of disease, compared to that in the non-laminitic controls. Although several markers, including *DEFB4B*, *S100A9*, *S100A8*, *PTGS2* and *MMP9*, were expressed in most or all severe acute samples, additional IL-17A target genes were detected in some, but not all of the severe acute cases examined (Figs 5 and 6). It is possible that the differences in expression of IL-17A target genes among the severe acute samples reflects variation in a specific gene's expression over time as the disease progresses, or as individual cells respond and adapt to receptor activation. There could also be an underlying genetic variability or pre-existing lamellar tissue damage among horses that affect the threshold for laminitis. Others previously observed "non-responders" to laminitis induction in experimental models [48, 49], consistent with variability among a cohort of individuals from a non-inbred, non-model organism. Additionally, we failed to detect significant expression of *CCL20* (Fig 6), perhaps indicating a difference in an IL-17A pathway response due to species or tissue differences. Overall, the detectable expression of 10 out of 11 downstream target genes in severe acute SLL tissue supports the hypothesis that lamellar tissue is responding via a similar IL-17A inflammatory pathway to that identified in human and animal models of auto-inflammatory diseases of the skin and extracutaneous sites, including psoriasis/psoriatic enthesis-arthritis and skin cancer, as well as the wound healing response in human or mouse skin [18, 19, 25].

Supporting the idea that lamellar tissue is responding to IL-17A-dependent signals, the receptor subunit, IL-17RA was expressed in lamellar tissues from both non-laminitic controls and in SLL samples (Fig 3), consistent with findings in human keratinocytes that the IL-17A receptor is constitutively expressed [28]. The mRNA for IL-17A was weakly detected, but more consistently detected in lamintic tissue samples compared to one positive among the non-laminitic controls. The weak detection is not surprising since we focused on gene expression, which does not capture protein levels either within cells or after secretion. In addition, the lamellar tissues examined showed low numbers of infiltrating immune cells that are the likely source of secreted IL-17A. Further studies are required to detect cell surface markers and IL-17A protein to determine which cell types are producing the cytokine and what is triggering IL-17A release within the lamellar environment to activate receptors and expression of downstream targets.

Whether activation of the IL-17A pathway in SLL is part of a mechanism driving disease progression or a downstream response to tissue damage is presently unknown. Supporting the idea that IL-17A-dependent inflammation contributes to disease is the observation that several IL-17A target genes examined here were upregulated at the developmental/subclinical or moderate acute stages. The most prominent gene was *DEFB4B*, which showed an ~2–10 fold increased expression level at developmental/subclinical and moderate acute stages compared to non-laminitic controls (Fig 4). *PTGS2* and *IL6* were also clearly expressed at the developmental/subclinical stage (S1 Fig). These data support the hypothesis that the IL-17A pathway is upregulated early in disease progression and could serve to amplify the response to IL-17A produced by inflammatory cells homing to stressed or dying cells, reducing epidermal function and contributing to loss of mechanical support.

The IL-17A pathway upregulation, detected here by expression of pathway target genes in natural cases of SLL, is also likely present in laminitis associated with other risk factors. In models of SRL, one or more target genes of the IL-17A pathway have been identified as genes upregulated after experimental induction of laminitis [5, 15, 43, 45, 48, 50–55]. In the hyperinsulinemia/euglycemic clamp model of EL, the cohort of genes examined included few downstream targets of IL-17A activation, but these studies identified both *MMP9* [56] and *TNFα* [57] upregulation. Several studies of naturally occurring cases of laminitis have described increased *MMP9* but the initiating trigger(s) in these natural cases was not described [55, 58]. Taken together, data from models of SRL and EL, as well as the results presented here for SLL, indicate that IL-17A based inflammation may be a shared feature among the three major triggers of laminitis, although this conclusion needs to be confirmed experimentally by a more comprehensive investigation of IL-17A levels and target gene expression.

In human psoriasis, a disease that has some clinical and histomorphological features similar to equine laminitis, keratinocytes of the skin express the IL-17A receptor, respond to IL-17A by expression of multiple IL-17A target genes, and are thought to make a major contribution to disease progression [18, 28, 30, 34]. We found that keratinocytes of the equine lamellae express both *DEFB4B* and calprotectin (S100A8/S100A9 dimer), as shown in Figs 7 and 8. Both *DEFB4B* and calprotectin were present in some, but not all, suprabasal keratinocytes. In equine laminitis, a major feature is progressive lamellar epidermal dysplasia characterized by acanthosis (areas with expansion of the suprabasal cell layers) and development of discernable desmosomal attachments [11], a morphological transformation resembling stratified squamous epidermis. Keratinocytes in these acanthotic regions also stained positively for the PAS histochemical stain that binds glycoproteins or glycogen (Figs 7 and 8). PAS-positive lamellar epithelial cells are not present in healthy adult lamellae [11] (S3 Fig). Whether PAS-positive staining is always correlated with *DEFB4B* or calprotectin expression has not been examined, but the co-localizations indicate that the same region of dysplastic lamellar epithelial cells is also responding to IL-17A receptor activation.

Several studies using experimental models of SRL also support a role of lamellar keratinocytes in producing IL-17A target genes. First, calprotectin was also localized to keratinocytes in a model of SRL [45]. Second, epithelial cells isolated by laser microdissection showed upregulation of several IL-17A pathway target genes measured by RNA-seq [15]. Although other cell types in the equine lamellae or DP may also express IL-17A target genes, given their abundance within the foot [59], the results from this study and others [15, 16, 43, 45, 48, 52–54, 57–60] suggest that keratinocytes are likely the major cell type expressing IL-17A target genes in SLL as well as other types of laminitis.

Assuming IL-17A pathway activation contributes to disease progression in equine laminitis, as it does in human skin diseases, blocking this pathway could lead to development of a therapeutic treatment amenable to use in horses. Inhibiting IL-17A signaling in the horse will not be as simple as applying human therapies since the most successful psoriasis treatments use monoclonal antibodies to block IL-17A receptor activation [21]; these antibodies may not bind the equine IL-17A receptor, may be antigenic in equines, and are likely cost-prohibitive. Local inhibition of IL-17A, possibly using an adeno-associated viral vector to deliver genes able to inhibit IL-17A-dependent signals, or to express inhibitory cytokines to block inflammation, as demonstrated recently [61], could be promising once it is established that the IL-17A pathway contributes to laminitis progression.

## Supporting information

S1 Fig Expression of 9 IL-17A target genes in developmental/subclinical, moderate acute, and severe chronic SLL disease stages detected by RT-PCR. *PTGS2*, *CCL2*, *TNFα* and *IL6* were expressed in some of the developmental/subclinical and moderate acute samples examined. *CCL20* was not detected at these disease stages. Expression of all 9 target genes was detected in the 5 severe chronic samples. Base pair lengths are indicated to the left of gels and sample numbers are indicated below each lane. *DEFB4B* expression at all disease stages is shown in Fig 4. Sample information is listed in Table 1.
(TIF)

S2 Fig Two additional SLL cases showing regional co-localizations of PAS positive cells, DEFB4B (purple product; in situ hybridization) and calprotectin (green; immunofluorescence). A. Severe acute case (Sample # 27). Boxed region for PASH and DEFB4B panels are shown below for calprotectin. Three PELs are marked in both the DEFB4B and calprotectin images. B. Severe chronic case (Sample # 38). Boxed region is shown in the bottom row and includes calprotectin localization (green). Sequential FFPE lamellar tissue sections were used for each label. The calprotectin images include rhodamine-tagged wheat germ agglutinin (red) as a counterstain to highlight extracellular matrix and cell membranes.
(TIF)

S3 Fig Non-laminitic control tissues do not express *DEFB4B* or calprotectin. Sequential FFPE lamellar tissue sections from sample #3 stained for PASH (left), in situ hybridization to localize *DEFB4B* expressing cells (middle) or calprotectin (green; right) (n = 3 using samples 1, 3 and 5). *DEFB4B* expression was not detected in sections from non-laminitic limbs as evidenced by the lack of purple color product. Tissue sections from non-laminitic limbs also do not show detectable calprotectin expression (green). Extracellular matrix and cell membranes are marked in red by the counterstain, rhodamine-tagged wheat germ agglutinin (WGA). The darker shadows in the rhodamine signal that appear as a window pane-like pattern are an artifact produced by stitching a series of images together (Methods). Images are oriented with the abaxial region is toward the top and the axial region toward the bottom. A higher magnification PASH image is shown to highlight PAS-positive basement membranes (arrow) and lack of PAS positive cells within SELs (asterisk).
(TIF)

S4 Fig Sense control probe does not hybridize to lamellar tissue. An additional section from the tissue shown in Figs 7 and 8 is shown after hybridization with a sense probe as a control for in situ hybridization. A low magnification composite for the anti-sense probe is shown to the left for comparison (this composite is also shown in Fig 7). Hybridization with the sense probe shows only dim purple reaction product. The boxed regions are shown at higher magnification after a 90° clockwise rotation. The small purple dots mark some cell nuclei, but this pattern is not observed with the anti-sense probe.
(TIF)

S1 Table Individual fold change values for *DEFB4B* and *S100A9* measured by qPCR. (DOCX)

S2 Table Pairwise comparisons of *DEFB4B* or *S100A9* gene expression fold changes by Wilcoxon-Mann-Whitney rank sum tests. (DOCX)

S1 File qPCR data. (XLSX)

S2 File Original images of all gels. (PDF)
