# Supplementary figures and images for: Interleukin-17A pathway target genes are upregulated in Equus caballus supporting limb laminitis
Source: PLoS One. 2020 Dec 10;15(12):e0232920. doi: 10.1371/journal.pone.0232920 (PMC7728170; doi:10.1371/journal.pone.0232920)

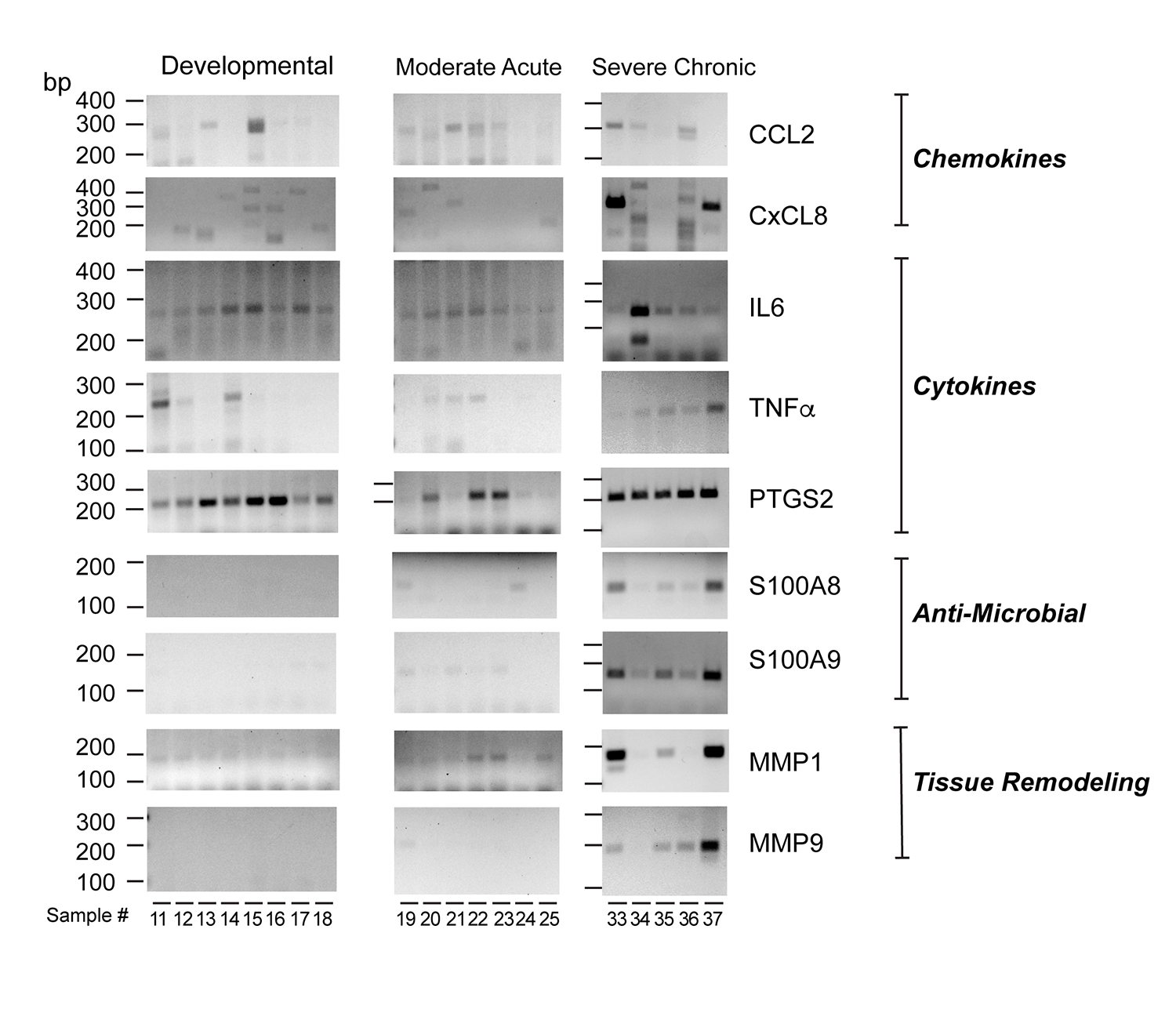

Supplement: S1 Fig — PTGS2, CCL2, TNFα and IL6 were expressed in some of the developmental/subclinical and moderate acute samples examined. CCL20 was not detected at these disease stages. Expression of all 9 target genes was detected in the 5 severe chronic samples. Base pair lengths are indicated to the left of gels and sample numbers are indicated below each lane. DEFB4B expression at all disease stages is shown in Fig 4. Sample information is listed in Table 1. (TIF) [file pone.0232920.s001.tif]

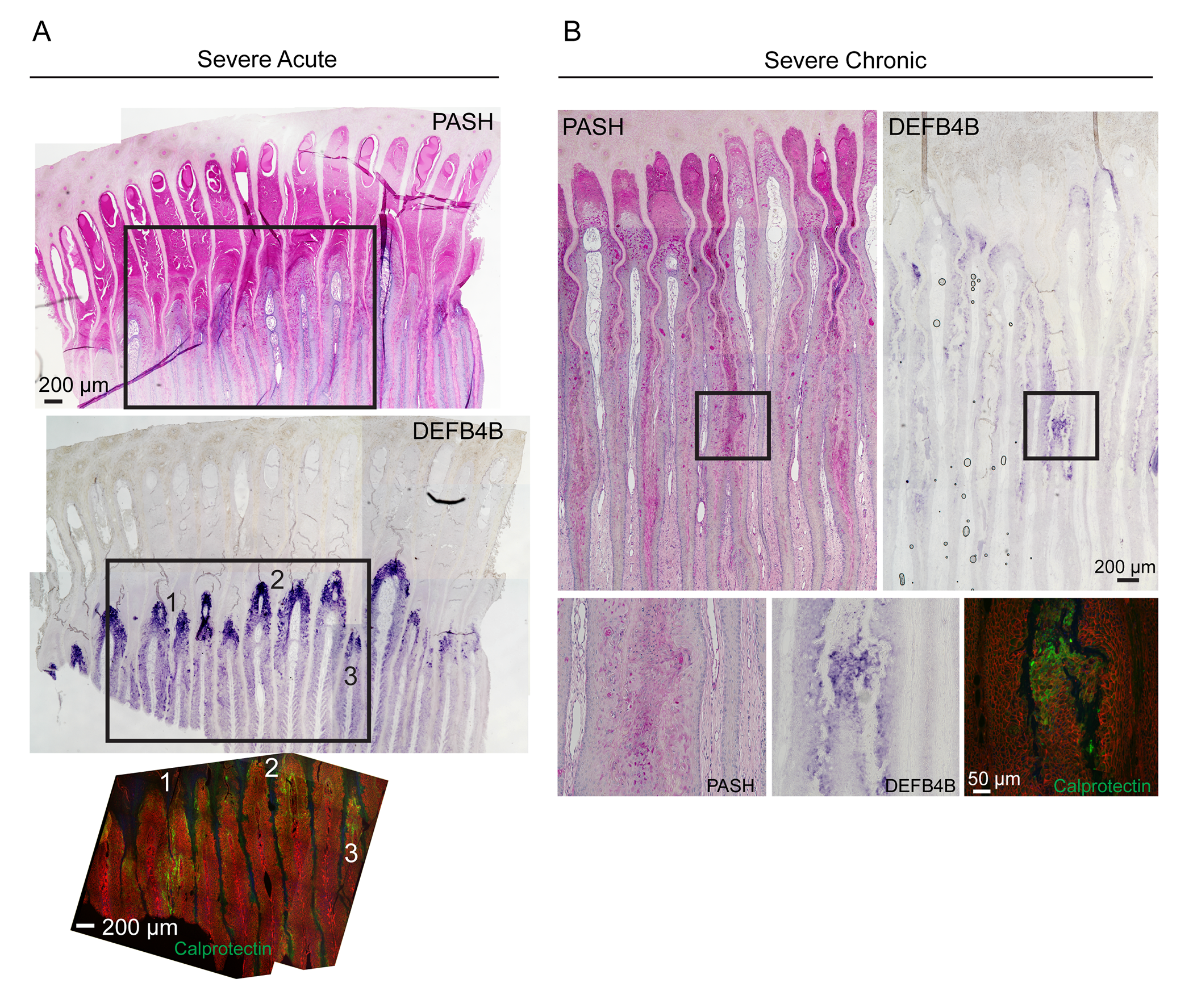

Supplement: S2 Fig — A. Severe acute case (Sample # 27). Boxed region for PASH and DEFB4B panels are shown below for calprotectin. Three PELs are marked in both the DEFB4B and calprotectin images. B. Severe chronic case (Sample # 38). Boxed region is shown in the bottom row and includes calprotectin localization (green). Sequential FFPE lamellar tissue sections were used for each label. The calprotectin images include rhodamine-tagged wheat germ agglutinin (red) as a counterstain to highlight extracellular matrix and cell membranes. (TIF) [file pone.0232920.s002.tif]

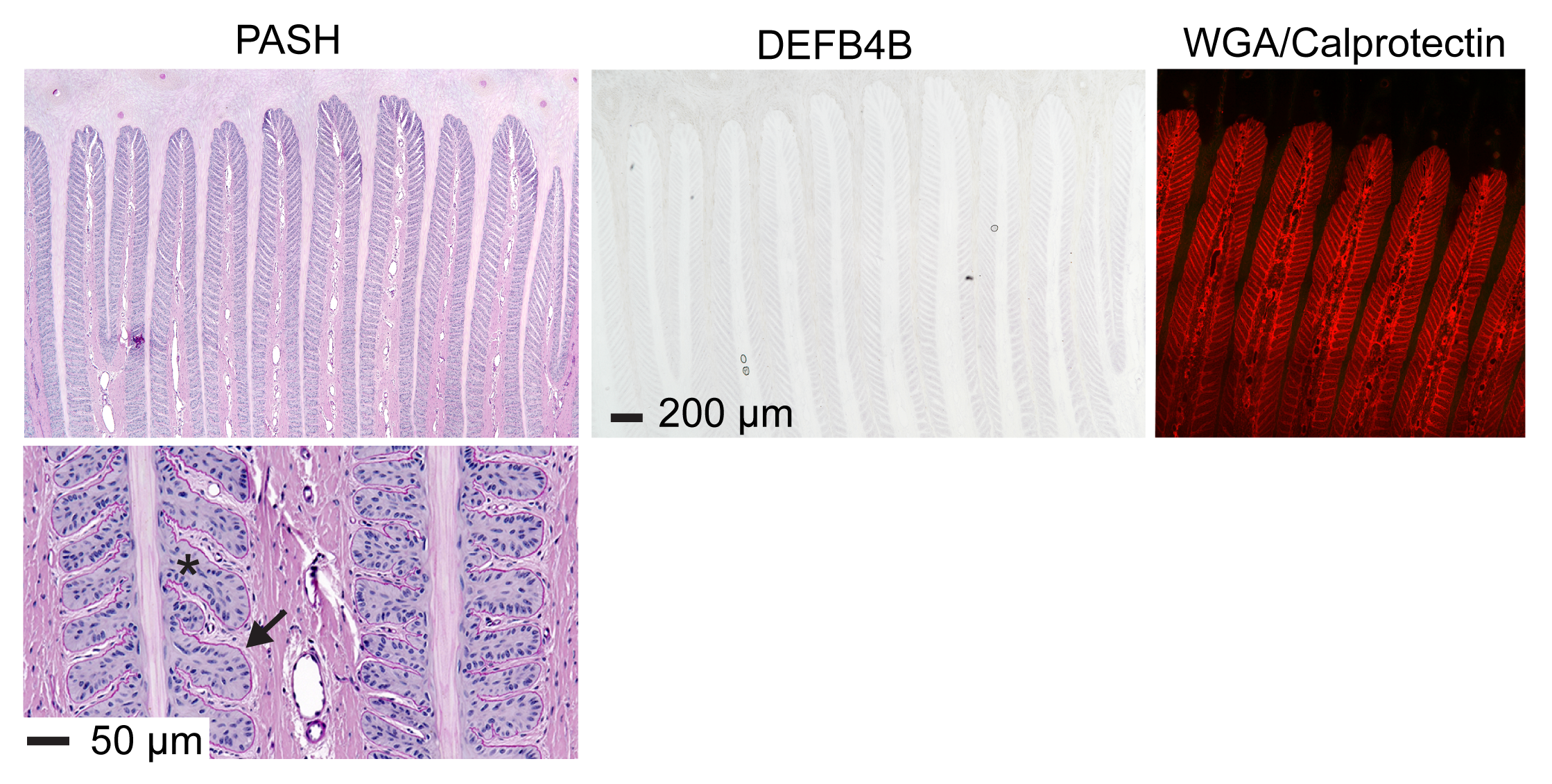

Supplement: S3 Fig — Sequential FFPE lamellar tissue sections from sample #3 stained for PASH (left), in situ hybridization to localize DEFB4B expressing cells (middle) or calprotectin (green; right) (n = 3 using samples 1, 3 and 5). DEFB4B expression was not detected in sections from non-laminitic limbs as evidenced by the lack of purple color product. Tissue sections from non-laminitic limbs also do not show detectable calprotectin expression (green). Extracellular matrix and cell membranes are marked in red by the counterstain, rhodamine-tagged wheat germ agglutinin (WGA). The darker shadows in the rhodamine signal that appear as a window pane-like pattern are an artifact produced by stitching a series of images together (Methods). Images are oriented with the abaxial region is toward the top and the axial region toward the bottom. A higher magnification PASH image is shown to highlight PAS-positive basement membranes (arrow) and lack of PAS positive cells within SELs (asterisk). (TIF) [file pone.0232920.s003.tif]

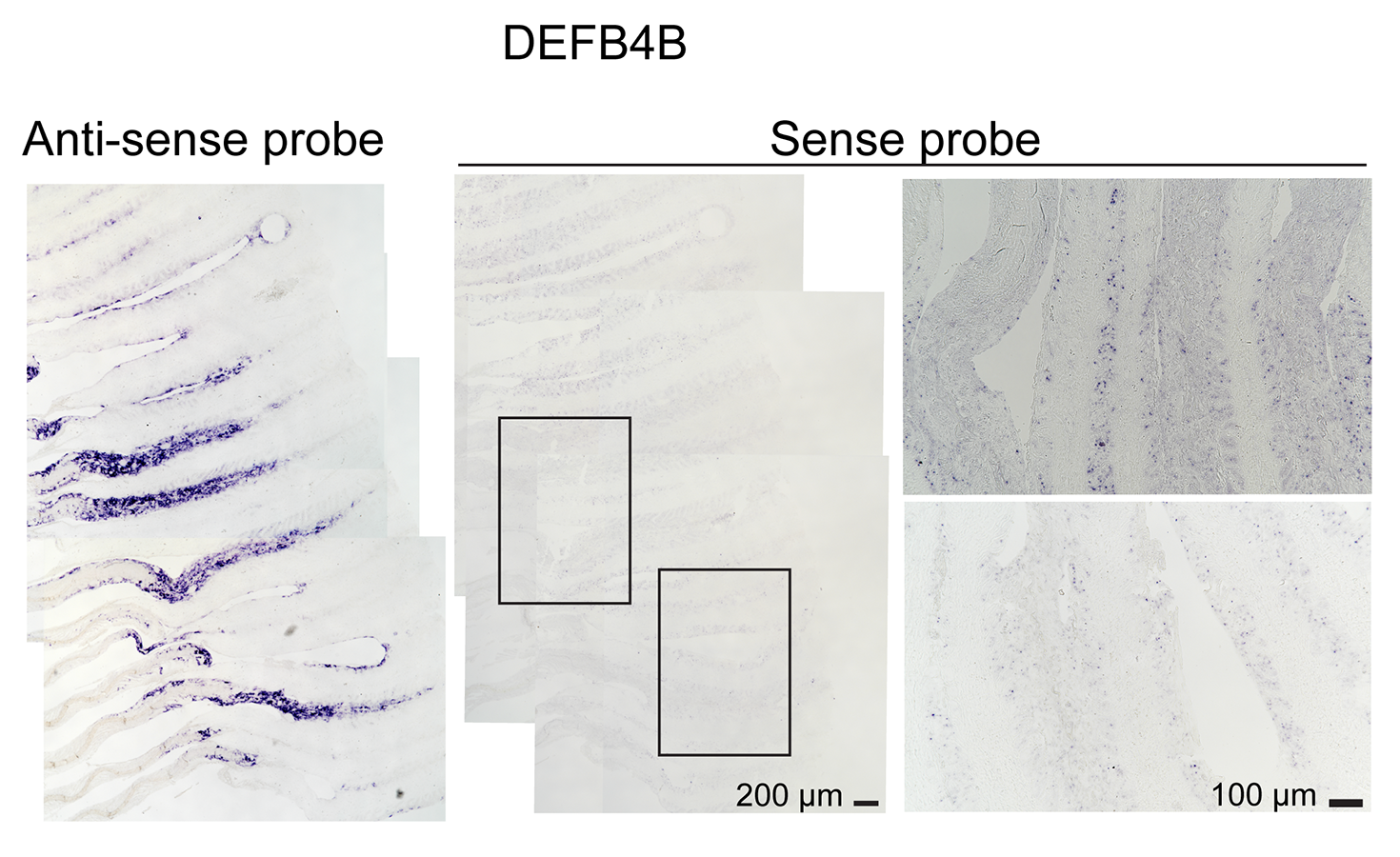

Supplement: S4 Fig — An additional section from the tissue shown in Figs 7 and 8 is shown after hybridization with a sense probe as a control for in situ hybridization. A low magnification composite for the anti-sense probe is shown to the left for comparison (this composite is also shown in Fig 7). Hybridization with the sense probe shows only dim purple reaction product. The boxed regions are shown at higher magnification after a 90° clockwise rotation. The small purple dots mark some cell nuclei, but this pattern is not observed with the anti-sense probe. (TIF) [file pone.0232920.s004.tif]
