# Supplementary material for: Interleukin-17A pathway target genes are upregulated in Equus caballus supporting limb laminitis
Source: PLoS One. 2020 Dec 10;15(12):e0232920. doi: 10.1371/journal.pone.0232920 (PMC7728170; doi:10.1371/journal.pone.0232920)
Supplement: S1 Table — (DOCX) [file pone.0232920.s005.docx]

S1 Table: *DEFB4B* and *S100A9* expression fold changes measured by qPCR

| Gene Expression Fold Changes | | | | | |
| --- | --- | --- | --- | --- | --- |
| Gene & Primer Pair | Non-Laminitic | Developmental/ subclinical | Moderate Acute | Severe Acute | Severe Chronic |
| *DEFB4B*  Primer Pair 1 | 1.00 | 3.46 | 3.60 | 15.38 | 207.93 |
|  | 0.48 | 1.51 | 6.11 | 37.53 | 51.69 |
|  | 0.23 | 10.24 | 2.02 | 18.43 | 107.04 |
|  | 0.32 | 5.18 | 0.64 | 107.40 | 14.75 |
|  | 1.46 | 9.57 | 1.88 | 14.77 | 14.75 |
|  | 1.53 | 5.16 | 1.11 | 64.39 |  |
|  | 0.67 | 2.56 | 2.71 | 18.44 |  |
|  | 0.49 | 2.15 |  |  |  |
|  |  |  |  |  |  |
| *DEFB4B*  Primer Pair 2 | 1.00 | 3.35 |  | 19.93 |  |
|  | 0.77 | 4.69 |  | 35.68 |  |
|  | 0.95 | 8.76 |  | 33.69 |  |
|  | 0.45 | 9.97 |  | 94.93 |  |
|  | 2.89 | 12.90 |  | 15.34 |  |
|  | 3.87 | 5.56 |  | 101.08 |  |
|  | 0.62 | 1.80 |  | 24.52 |  |
|  |  | 2.65 |  |  |  |
|  |  |  |  |  |  |
| *S100A9* | 1.00 |  |  | 15.83 |  |
|  | 1.36 |  |  | 18.51 |  |
|  | 5.14 |  |  | 17.23 |  |
|  | 1.2 |  |  | 429.00 |  |
|  | 0.925 |  |  | 38.75 |  |
|  | 1.65 |  |  | 56.54 |  |
|  | 2.22 |  |  | 42.63 |  |
|  | 0.97 |  |  |  |  |

Fold changes in gene expression levels were calculated relative to Sample #1 as described in Methods.
