# Supplementary material for: Interleukin-17A pathway target genes are upregulated in Equus caballus supporting limb laminitis
Source: PLoS One. 2020 Dec 10;15(12):e0232920. doi: 10.1371/journal.pone.0232920 (PMC7728170; doi:10.1371/journal.pone.0232920)
Supplement: S2 Table — (DOCX) [file pone.0232920.s006.docx]

S2 Table: Pairwise Comparisons of *DEFB4B* or *S100A9* gene expression fold changes by Wilcoxon-Mann-Whitney Rank Sum Tests

|  | Disease Stage | | | | |
| --- | --- | --- | --- | --- | --- |
| Gene & Primer Pair |  | Developmental/ subclinical | Moderate Acute | Severe Acute | Severe Chronic |
| *DEFB4B*  Primer Pair 1 | Non-Laminitic | p=0.0003 | p=0.0093 | p=0.0003 | p=0.0043 |
|  | Developmental/ subclinical |  | p=0.152 | p=0.0003 | p=0.0043 |
|  | Moderate Acute |  |  | p=0.00058 | p=0.0057 |
|  | Severe Acute |  |  |  | p=1.00 |
|  |  |  |  |  |  |
| *DEFB4B*  Primer Pair 2 | Non-Laminitic | p=0.0059 |  | p=0.0003 |  |
|  | Developmental/ subclinical |  |  | p=0.0003 |  |
|  |  |  |  |  |  |
| *S100A9* | Non-Laminitic |  |  | p=0.0003 |  |

*DEFB4B* expression fold changes were first compared by Kruskal-Wallis rank sum tests to compare > 2 groups. For *DEFB4B* primer pair #1, comparison between all sample groups yielded a p value of <0.0001. For *DEFB4B* primer pair #2, comparison between 3 sample groups yielded a p value of 0.00013. Wilcoxon-Mann-Whitney rank sum tests were used for post hoc analyses and the pairwise p values are given in the table.
