# Supplementary material for: Interleukin-17A pathway target genes are upregulated in Equus caballus supporting limb laminitis
Source: PLoS One. 2020 Dec 10;15(12):e0232920. doi: 10.1371/journal.pone.0232920 (PMC7728170; doi:10.1371/journal.pone.0232920)
Supplement: S2 File — (PDF) [file pone.0232920.s008.pdf]

Fig 3: Original gels from RT-PCR amplifying *IL17RA* and *IL17A*. Samples are marked as listed in Table 1.

IL17RA

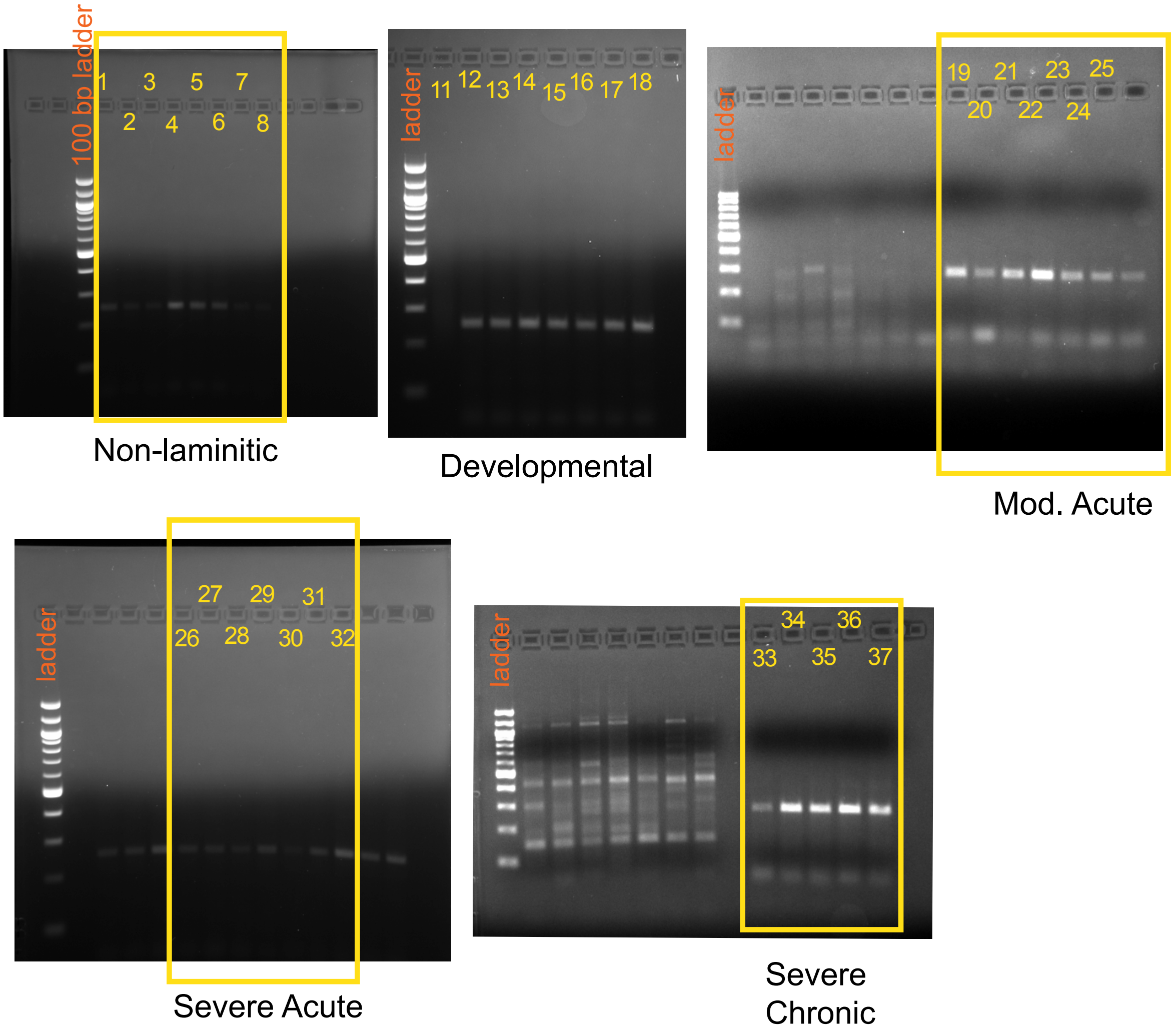

IL17A

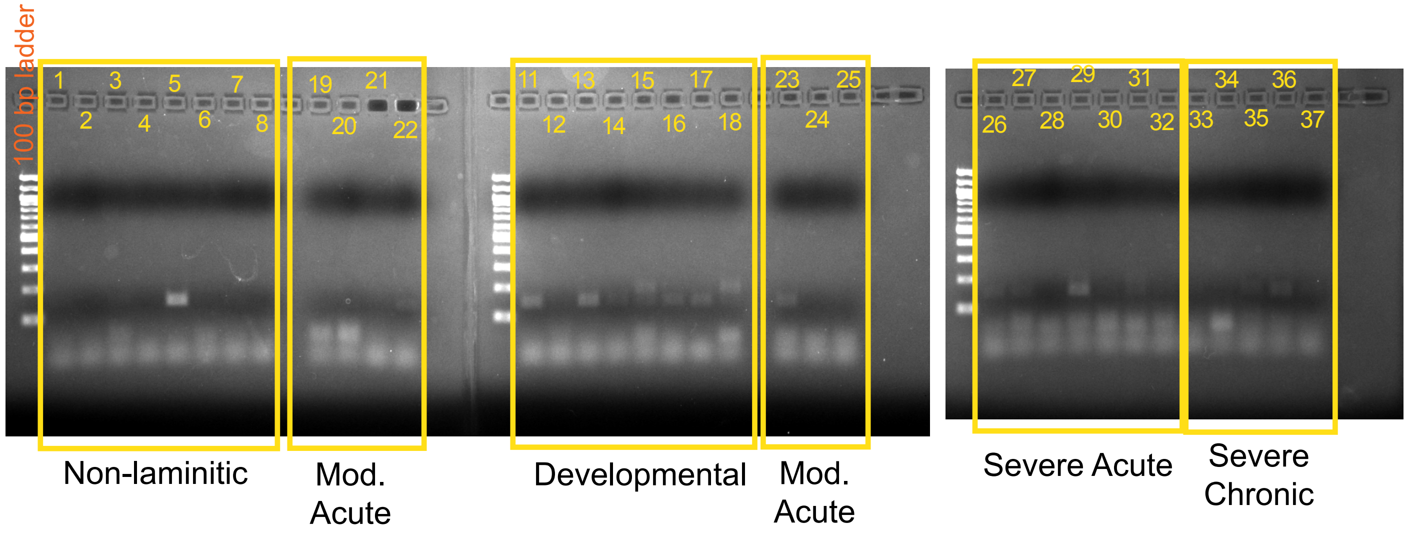

Fig 4: Original gels from qPCR end points after amplifying *DEFB4B or RACK1*. Samples are marked as listed in Table 1.

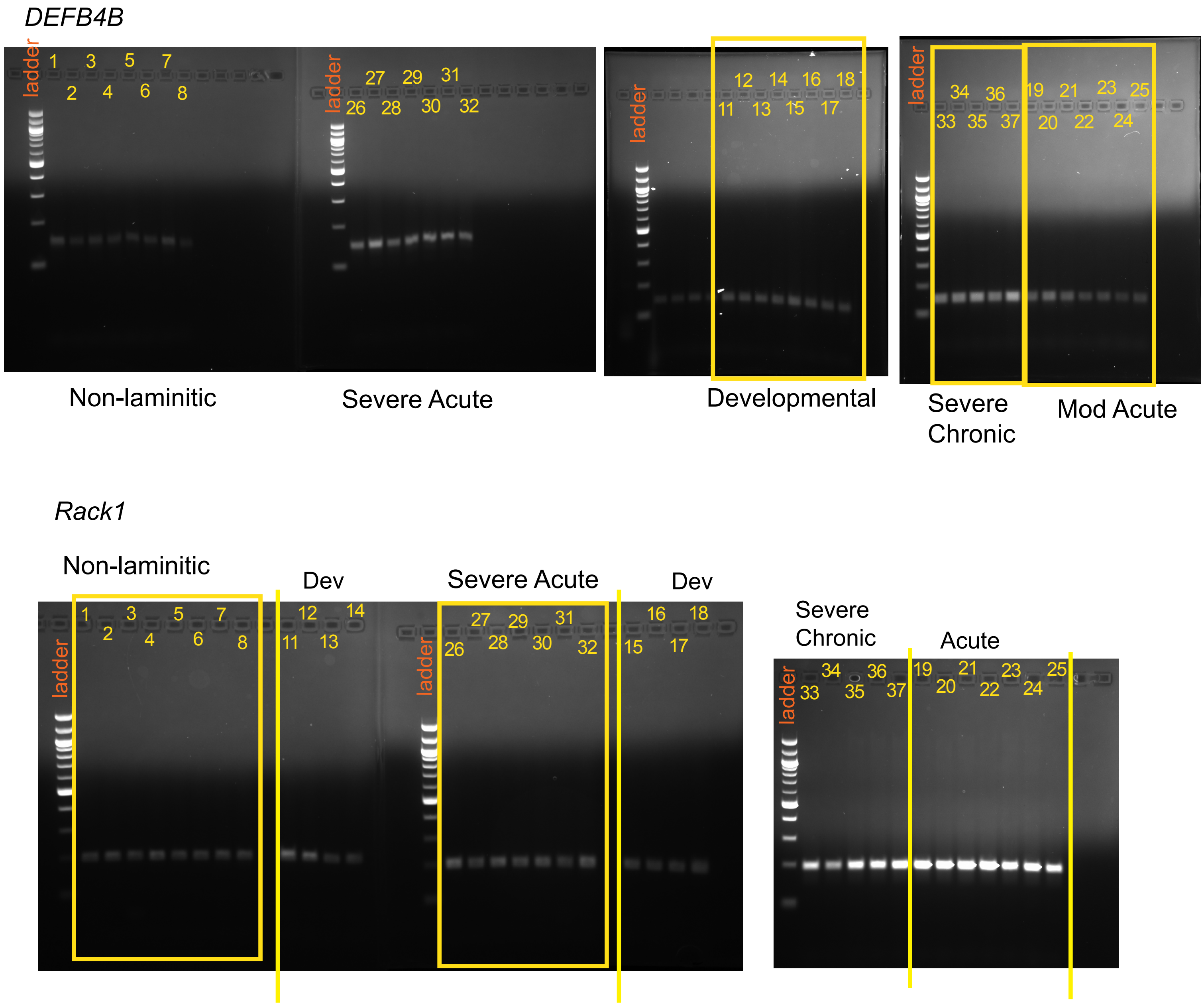

Fig 5A: Original gels of amplimers from RT-PCR or qPCR end points. Predicted amplimer sizes marked by yellow arrows. Samples are marked as listed in Table 1.

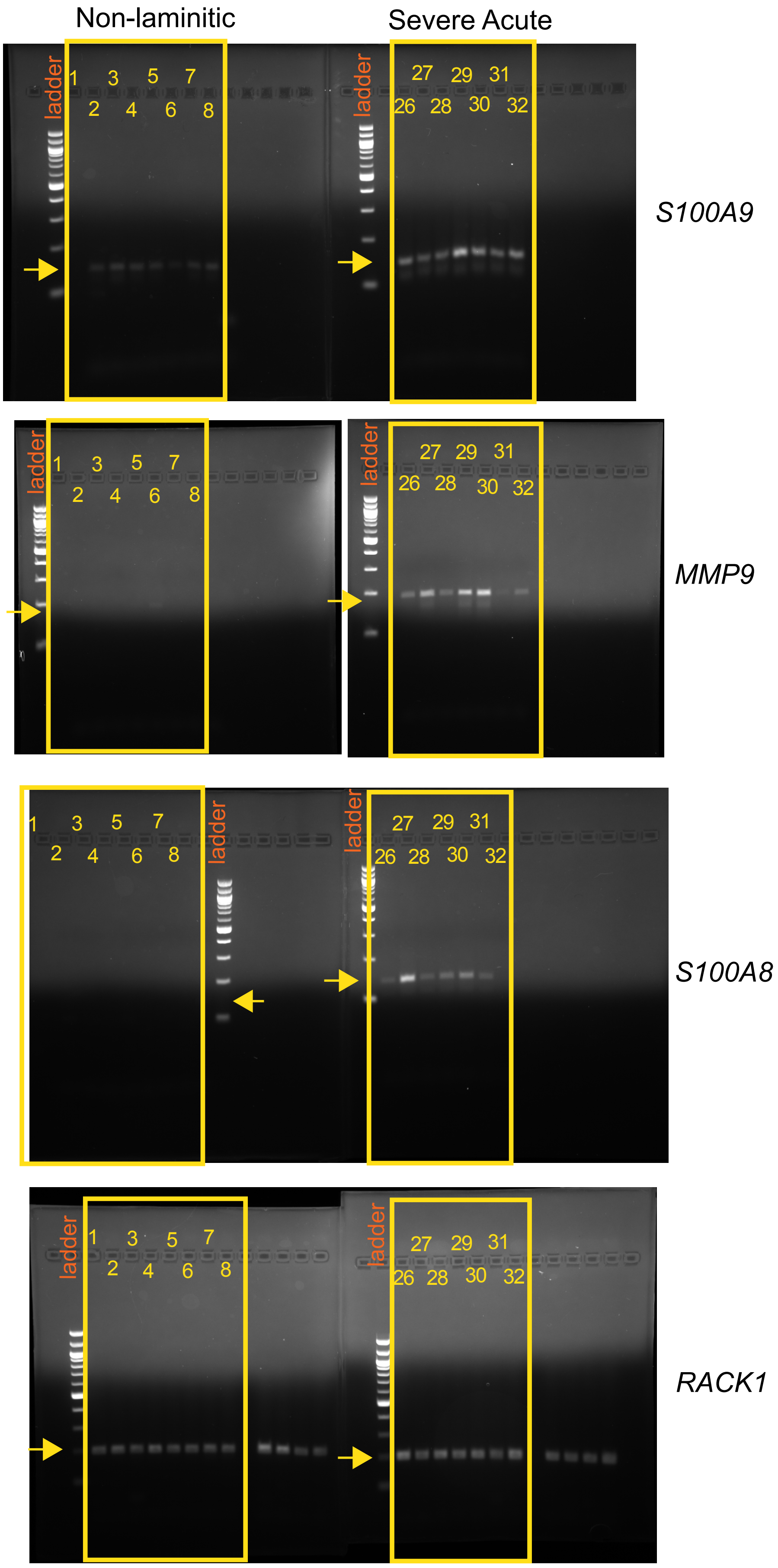

Fig 5A continued: Original gels of amplimers from RT-PCR or qPCR end points.
Predicted amplimer sizes marked by yellow arrows. Samples are marked as listed in Table 1.

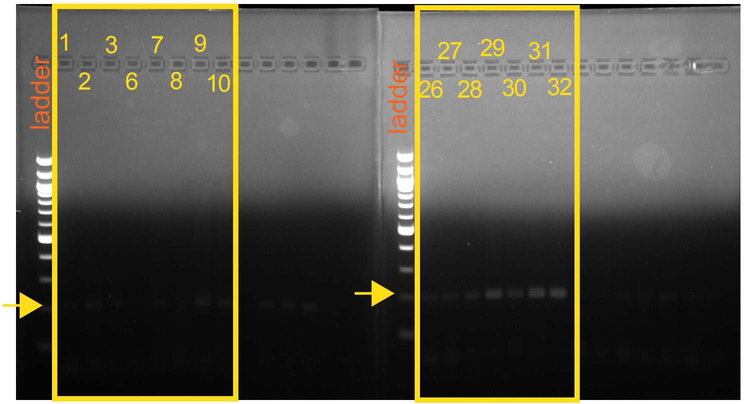

PTGS2

Fig 6A: Original gels of amplimers from RT-PCR. Samples are marked as listed in Table 1.

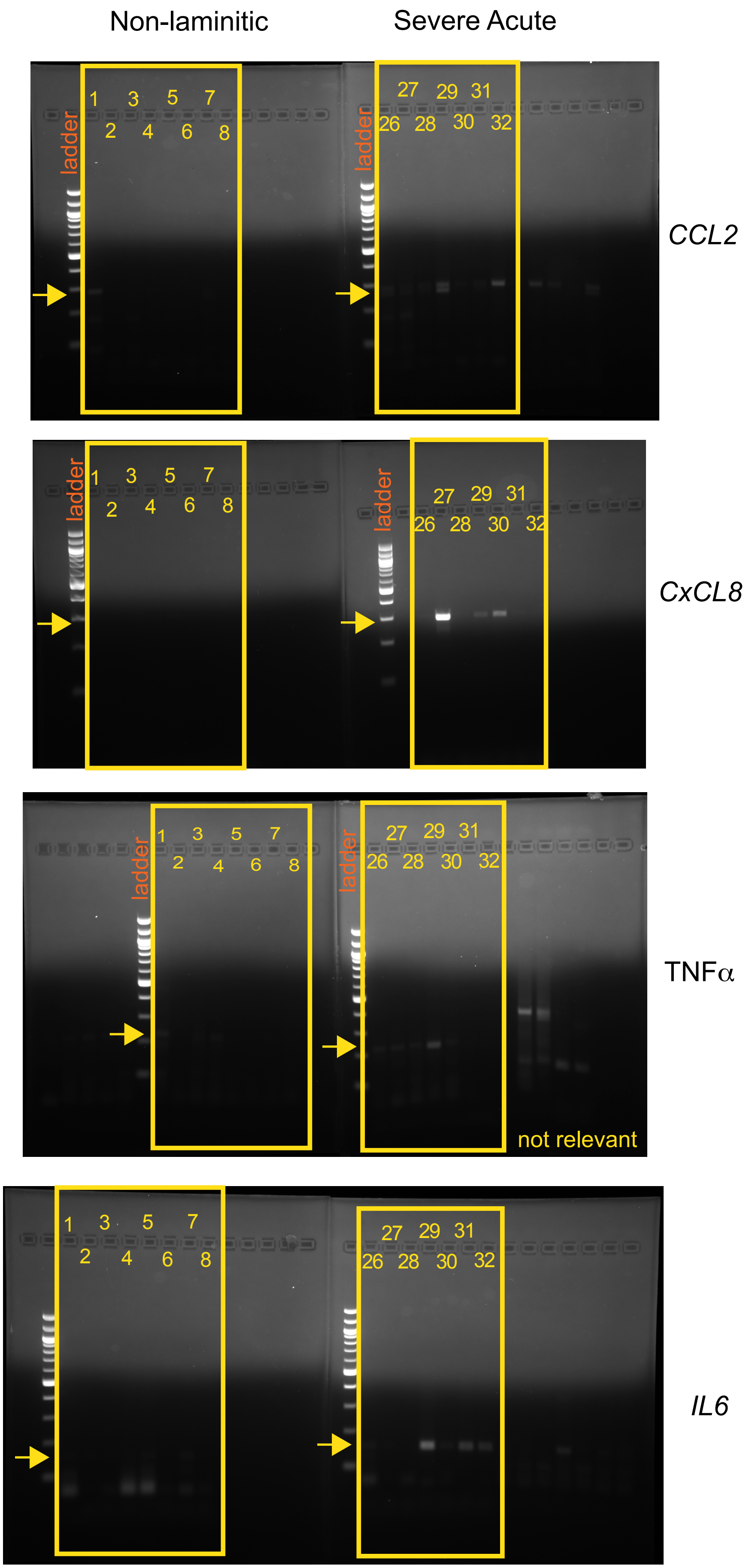

Fig 6A: Original gels continued. Expected amplimer sizes marked by yellow arrows.

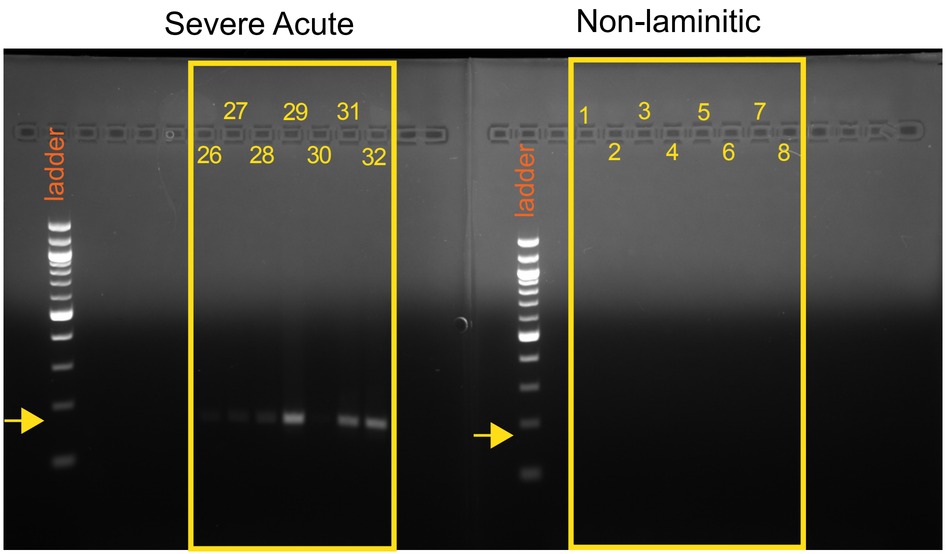

*Note gels are in reverse order here

MMP1

Fig 6B: Original gel

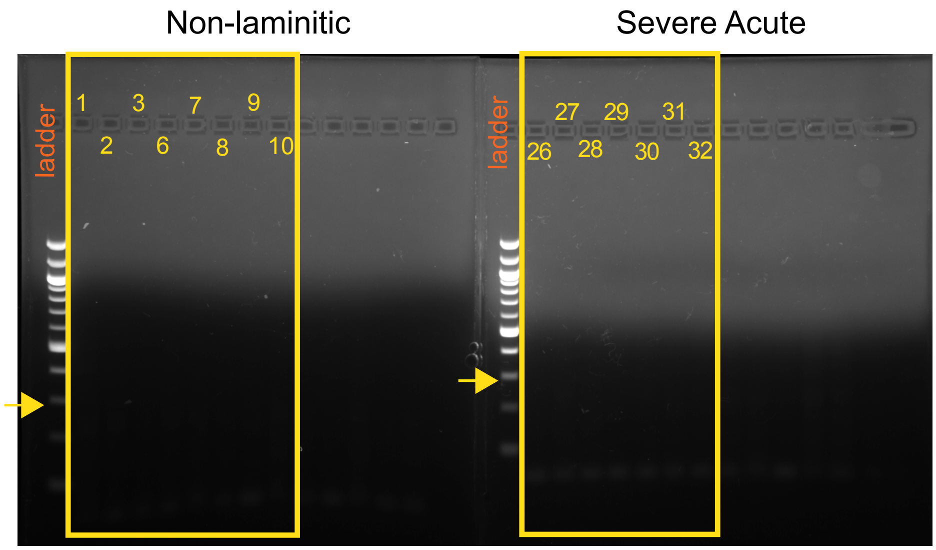

CCL20

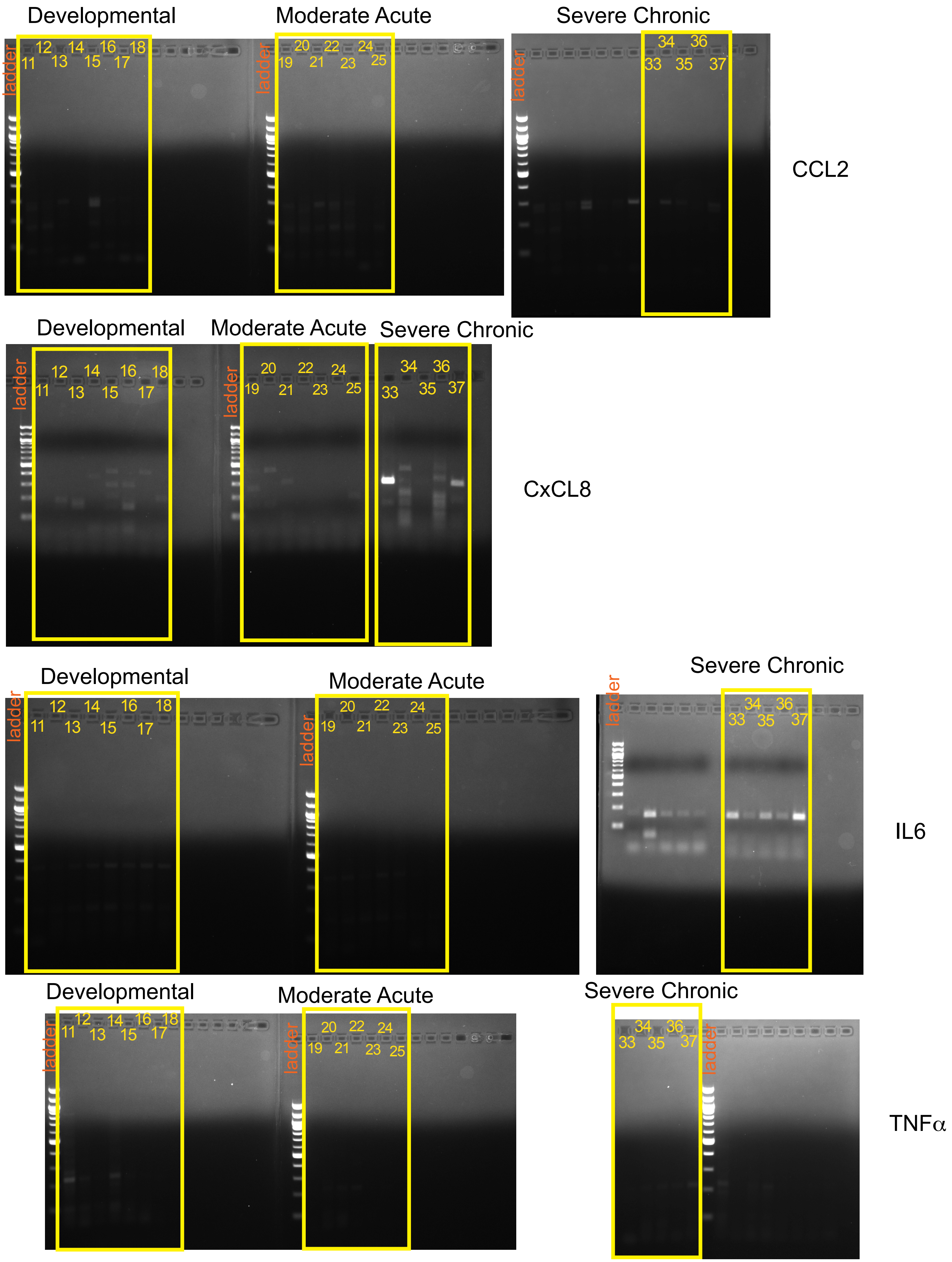

Developmental
Moderate Acute
Severe Chronic
ladder
11 12 13 14 15 16 17 18
19 20 21 22 23 24 25
33 34 35 36 37
CCL2
CxCL8
IL6
TNFα

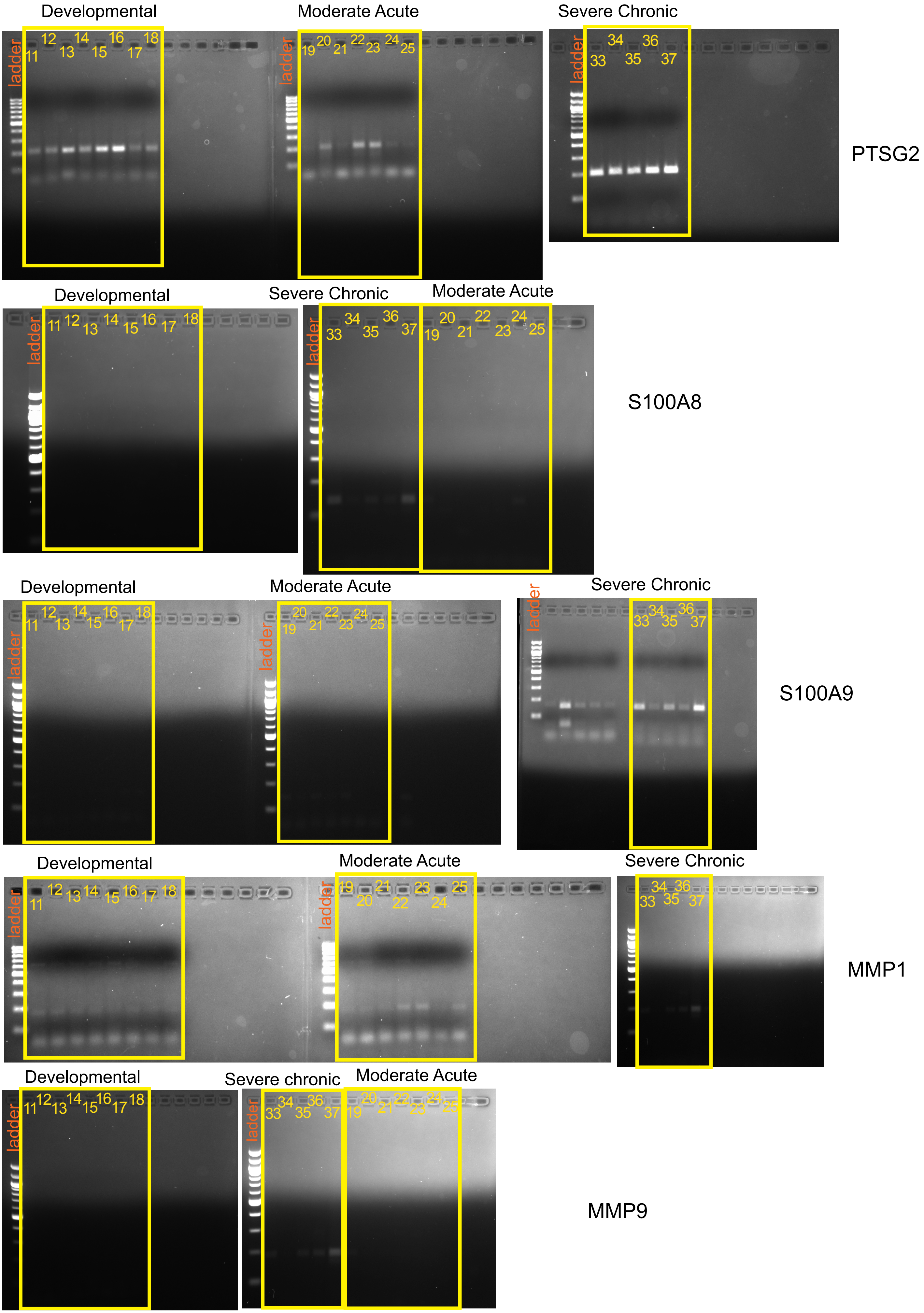
Developmental
Moderate Acute
Severe Chronic
ladder
11 12 13 14 15 16 17 18
19 20 21 22 23 24 25
33 34 35 36 37
PTSG2
Developmental
Severe Chronic
Moderate Acute
S100A8
Developmental
Moderate Acute
Severe Chronic
S100A9
Developmental
Moderate Acute
Severe Chronic
MMP1
Developmental
Severe chronic
Moderate Acute
MMP9
